# Distinct checkpoint and homolog biorientation pathways regulate meiosis I in *Drosophila* oocytes

**DOI:** 10.1371/journal.pgen.1011400

**Published:** 2025-01-29

**Authors:** Joanatta G. Shapiro, Neha Changela, Janet K. Jang, Jay N. Joshi, Kim S. McKim

**Affiliations:** Waksman Institute, Rutgers, the State University of New Jersey, Piscataway, New Jersey, United States of America; Indian Institute of Science Education and Research Mohali, INDIA

## Abstract

Mitosis and meiosis have two mechanisms for regulating the accuracy of chromosome segregation: error correction and the spindle assembly checkpoint (SAC). We have investigated the function of several checkpoint proteins in meiosis I of *Drosophila* oocytes. Increased localization of several SAC proteins was found upon depolymerization of microtubules by colchicine. However, unattached kinetochores or errors in biorientation of homologous chromosomes do not induce increased SAC protein localization. Furthermore, the metaphase I arrest does not depend on SAC genes, suggesting the APC is inhibited even if the SAC is not functional. Two SAC proteins, ROD of the ROD-ZW10-Zwilch (RZZ) complex and MPS1, are also required for the biorientation of homologous chromosomes during meiosis I, suggesting an error correction function. Both proteins aid in preventing or correcting erroneous attachments and depend on SPC105R for localization to the kinetochore. We have defined a region of SPC105R, amino acids 123–473, that is required for ROD localization and biorientation of homologous chromosomes at meiosis I. Surprisingly, ROD removal from kinetochores and movement towards spindle poles, termed “streaming,” is independent of the dynein adaptor Spindly and is not linked to the stabilization of end-on attachments. Instead, meiotic RZZ streaming appears to depend on cell cycle stage and may be regulated independently of kinetochore attachment or biorientation status. We also show that Spindly is required for biorientation at meiosis I, and surprisingly, the direction of RZZ streaming.

## Author summary

The spindle assembly checkpoint (SAC) is known to delay cell cycle progression until chromosomes are properly attached to microtubules. Meiotic cells often have modified cell cycle phases and natural arrest points such as metaphase I in *Drosophila*. We show that in *Drosophila* oocytes, SAC protein localization increases in response to loss of microtubules, but does not increase in response to a variety of kinetochore attachment errors. Thus, the function of the SAC appears to be limited to monitoring oocyte spindle assembly, and not required for accurate chromosome segregation. However, two of the SAC genes, *rod* and *Mps1*, are required for the biorientation of homologous chromosomes during meiosis I, suggesting an error correction function. ROD is part of the RZZ complex and is notable for its property of streaming off the kinetochores. However, our results show that streaming off the kinetochore may not contribute to RZZ regulation of microtubule attachments, and only be associated with SAC function. Instead, the establishment of stable end-on attachments may occur while RZZ is still present at kinetochores. We suggest that RZZ interacts with multiple motors to promote bidirectional movement of kinetochores along microtubules, which allows chromosomes to find and attach to the correct pole.

## Introduction

Errors during meiotic chromosome segregation can result in inviable gametes leading to spontaneous abortions and infertility, or viable offspring with chromosomal abnormalities. Several mechanisms exist in dividing cells to prevent aneuploidy resulting from improper kinetochore-microtubule (KT-MT) attachments. The spindle assembly checkpoint (SAC) delays anaphase onset until attachments form between kinetochores and microtubules [1,2]. Unattached kinetochores generate a signal involving SAC proteins such as MAD1, MAD2, BUB1, and BUB3 that have a global effect on all chromosomes and cell cycle progression. In contrast, error correction is specific to individual kinetochores whereupon interactions between microtubules and properly bi-oriented kinetochores are stabilized while those with improperly oriented kinetochores are weakened [3]. Multiple models have been proposed to explain how improperly oriented kinetochores are identified, involving both tension-dependent and -independent mechanisms, and how they are corrected, including phosphorylation of kinetochore proteins to destabilize attachments [4,5].

Error rates are higher in human oocytes than in spermatocytes, which has been attributed to several causes [6–8]. One hypothesis is that the SAC is weak or inefficient [7,9–11]. Indeed, *Xenopus* oocytes may not have a functional SAC [12,13]. The observation that mammalian oocytes progress through anaphase with segregation errors may be a result of a weak checkpoint [14,15]. The reason for a weak SAC in oocytes is not known, although possible contributing factors include the large size of oocytes or the lack of centrosomes [9,16–19]. The SAC may also have other functions in oocytes, such as regulating the development of the oocytes and the timing of meiotic progression [1,2,10].

*Drosophila* oocytes are an excellent model for studying meiosis in females, but the SAC has not been examined. *Drosophila* oocytes have kinetochores that include the KMN complex (KNL1-MIS12-NDC80) [20]. Biorientation of homologous chromosomes involves two types of kinetochore-microtubule attachments, lateral attachments that depend on KNL1/SPC105R and end-on attachments that depend on NDC80 [21,22]. To understand the contributions of SAC and error correction to biorientation in *Drosophila* oocytes, we characterized SAC protein localization during the first meiotic division, investigated how the SAC proteins are recruited to the kinetochores, and tested several SAC genes for functions in meiotic error correction. Our data suggest that the SAC is not sensitive to kinetochore attachment status and might not be required for the metaphase I arrest. Furthermore, among seven SAC proteins, only two, ROD of the ROD-ZW10-Zwilch (RZZ) complex and MPS1, are required for biorientation of meiotic homologous chromosomes at metaphase I.

We examined the role of RZZ in *Drosophila* oocytes because it has not been studied before in meiosis. Previous studies in *C*. *elegans* embryos and *Drosophila* neuroblasts suggest that RZZ prevents errors by inhibiting the premature stabilization of interactions between the ends of microtubules and NDC80 [23,24]. Furthermore, removal of RZZ from the kinetochores could alleviate the inhibition of end-on attachments. Indeed, RZZ components move along microtubules towards the spindle poles, a localization pattern termed “streaming”, in both mitotic cells [24–26] and oocytes [21,27]. We have shown that the RZZ complex is required for accurate homolog biorientation in meiosis I, which could indicate a role for the RZZ complex in delaying the formation of end-on attachments. We also show that Spindly, a dynein adaptor for RZZ, is required for homolog biorientation in meiosis I. However, RZZ streaming in oocytes is not required for, nor does it depend on, the formation of end-on attachments. In agreement with a previous study [27], we propose that RZZ streaming is initiated by a developmentally programmed metaphase I arrest, rather than lack of attachment errors. Biorientation, however, depends on an interaction between RZZ, Spindly, and possibly other motor activities that regulate chromosome movement and attachment status.

## Results

### Analysis of the SAC protein localization in oocytes

**Fig 1:**
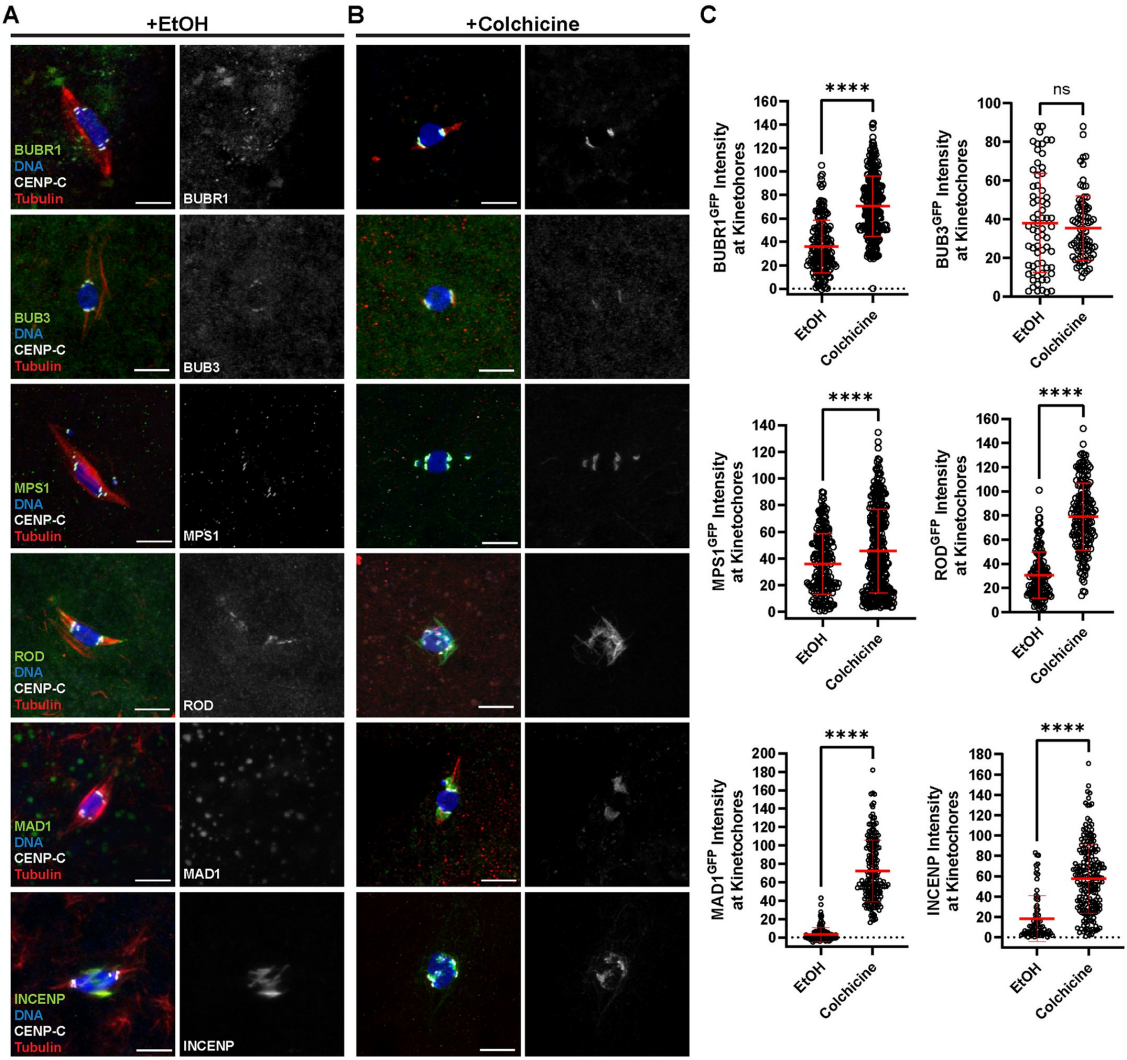
Localization of SAC proteins in oocytes. (A, B) Oocytes were incubated for one hour in Robb’s buffer with 0.5% EtOH (A) or 250 μM colchicine (B). BUBR1, BUB3, MPS1, ROD, and MAD1 were detected using GFP-tagged transgenes, while INCENP was detected using an anti-INCENP antibody (all proteins shown in green), DNA is in blue, CENP-C in white, and tubulin in red. Single channel images show SAC protein localization. All images are maximum intensity projections of z stacks. Scale bars represent 5 μm. (C) Quantification of SAC protein intensity at kinetochores, normalized to background signal (n for EtOH and colchicine = 313 and 174 for BUBR1, 67 and 83 for BUB3, 219 and 356 for MPS1, 139 and 184 for ROD, 138 and 185 for MAD1, and 74 and 200 for INCENP). Error bars show mean ± s.d.; ****P<0.0001, ns = no significance (unpaired two-tailed t test).

The localization of SAC proteins was examined in *Drosophila* oocytes. Mature *Drosophila* oocytes, known as stage 14, arrest at metaphase I with the chromosomes clustered together in a single mass, or karyosome, and the centromeres of homologous chromosomes oriented towards opposite poles. Several SAC proteins were detected at kinetochores in stage 14 oocytes, including BUBR1, BUB3, MPS1, and ROD (Fig 1A). In addition to its kinetochore localization, ROD localized to the spindle region between the pole and the centromeres (Fig 1). This redistribution of ROD from the kinetochores to the spindle is consistent with behavior previously observed in mitotic cells termed “streaming” [25,28,29]. Another RZZ subunit, ZW10 tagged with HA, overlapped with ROD^GFP^ staining, validating that ROD^GFP^ provides an accurate representation of the RZZ complex’s localization (S1A Fig).

In contrast to the above proteins, we did not detect MAD1 in stage 14 oocytes (Fig 1A). Therefore, we attempted to increase SAC protein localization by incubating stage 14 oocytes in colchicine for 60 minutes to remove nearly all microtubules. Following this treatment, robust kinetochore localization of MAD1 was observed in all oocytes (Fig 1B). Because colchicine induced a change in MAD1 localization, we tested if the localization of other checkpoint protein was increased by loss of microtubules. BUBR1 and MPS1 intensity increased in colchicine, suggesting their levels are sensitive to the SAC (Fig 1C). We did not, however, observe an increase in BUB3 localization.

A more dramatic response was observed with ROD. In colchicine-treated oocytes, ROD expanded around the kinetochores (Fig 1B). In some cases, ROD expanded to surround the karyosome and individual kinetochore foci could no longer be observed. This behavior of the RZZ complex likely corresponds to expansion of the fibrous corona, observed in miotic cells that lack kinetochore-microtubule (KT-MT) attachments [25,30]. This expansion is thought to facilitate microtubule capture by increasing the surface area of the kinetochore which can contact the microtubules. Thus, in *Drosophila* meiotic oocytes, the fibrous corona expands when microtubules are absent.

Activation of the SAC is also associated with elevated Aurora B activity [31, 32]. In *Drosophila* oocytes, the chromosomal passenger complex (CPC) is recruited to the chromosomes but then moves to the central spindle in prometaphase I oocytes [33]. CPC proteins are not detected at the meiotic centromeres in fixed samples [33,34]. In colchicine-treated oocytes, however, the CPC component INCENP localized to the chromosomes and the centromeres (Fig 1B and 1C). These results suggest that, like MAD1, colchicine treatment causes increased localization of the CPC to the centromeres.

### Effect of kinetochore-microtubule attachments on SAC protein localization

**Fig 2:**
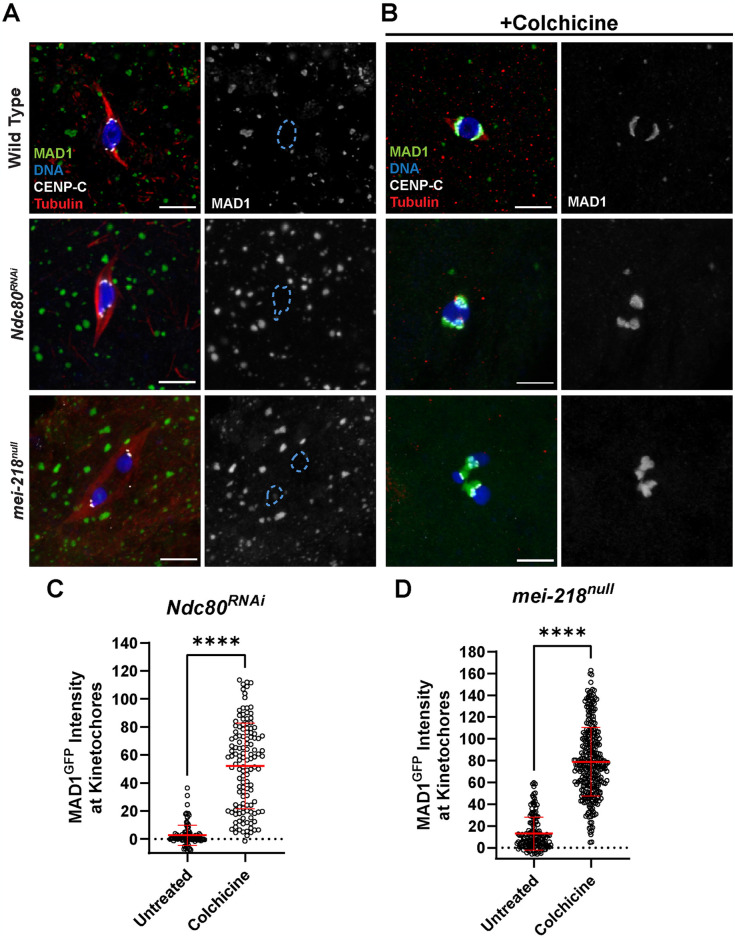
Induction of MAD1 localization is independent of tension and attachment status. (A, B) MAD1^GFP^ localization in wild-type, *Ndc80*^*RNAi*^, or *mei-218*^*null*^ oocytes that were either untreated (A) or treated with 250 μM colchicine (B). MAD1^GFP^ is shown in green, DNA in blue, CENP-C in white, and tubulin in red. Single channel images (right) show MAD1^GFP^ with karyosome outlines in dashed blue in (A). All images are maximum intensity projections of z stacks. Scale bars represent 5 μm. (C, D) Quantification of MAD1^GFP^ intensity at kinetochores, normalized to background GFP signal (n = 106 and 127 kinetochores for (C) and 158 and 311 kinetochores for (D)). Error bars show mean ± s.d.; ****P<0.0001 (unpaired two-tailed t test).

In all colchicine-treated oocytes, MAD1 localization was consistent and observed on all kinetochores, suggesting a global increase in SAC protein localization in response to the absence of microtubules. To investigate the factors that regulate SAC localization in oocytes, we examined MAD1 in conditions where microtubule attachments to kinetochores or tension were disrupted. NDC80 is required for end-on attachments of kinetochores to microtubules, so nearly all attachments in *Ndc80*^*RNAi*^ oocytes are lateral [22] (S2A Fig). MAD1 was not detected in *Ndc80*^*RNAi*^ oocytes unless they were treated with colchicine, suggesting that the SAC is insensitive to the loss of end-on attachments (Fig 2A–2C). We also examined MAD1 in a *mei-218* mutant (*mei-218*^*null*^), which has a high error rate and lacks tension on the kinetochores due to the absence of chiasmata [35]. MAD1 was not detected in *mei-218*^*null*^ oocytes unless they were treated with colchicine, suggesting that loss of tenson does not induce an increase in SAC protein localization (Fig 2A, 2B and 2D). The absence of MAD1 in the untreated *mei-218*^*null*^ oocytes is consistent with the prior observation that oocytes lacking chiasma bypass the metaphase I arrest and precociously enter anaphase in stage 14 oocytes [35]. These results suggest that SAC proteins localization increases in the absence of microtubules but not due to biorientation errors, or to the lack of stable attachments or tension.

### Localization of checkpoint proteins in *Drosophila* oocytes depends on SPC105R

The outer kinetochore protein KNL1/SPC105R is involved in the recruitment of several checkpoint proteins in mitotic cells [36,37]. Therefore, we investigated the role of SPC105R, the *Drosophila* homolog of KNL1, in the recruitment of MAD1, ROD, MPS1, and the CPC to kinetochores in *Drosophila* oocytes (S1 Table). To test if SPC105R is required for the recruitment of INCENP and MAD1, we treated *Spc105R*^*RNAi*^ oocytes with colchicine. Colchicine-induced kinetochore localization of both INCENP and MAD1 depended on SPC105R (S3A–S3D Fig). However, both still localized to the chromosomes. In the absence of SPC105R, MAD1 moved to the pericentric regions adjacent to the centromeres, while INCENP localized to all chromatin and occasionally to regions adjacent to the chromatin. The observation that MAD1 localized in colchicine-treated oocytes depleted of SPC105R suggests that the oocyte can respond to the loss of microtubules even in the absence of the outer kinetochore.

**Fig 3:**
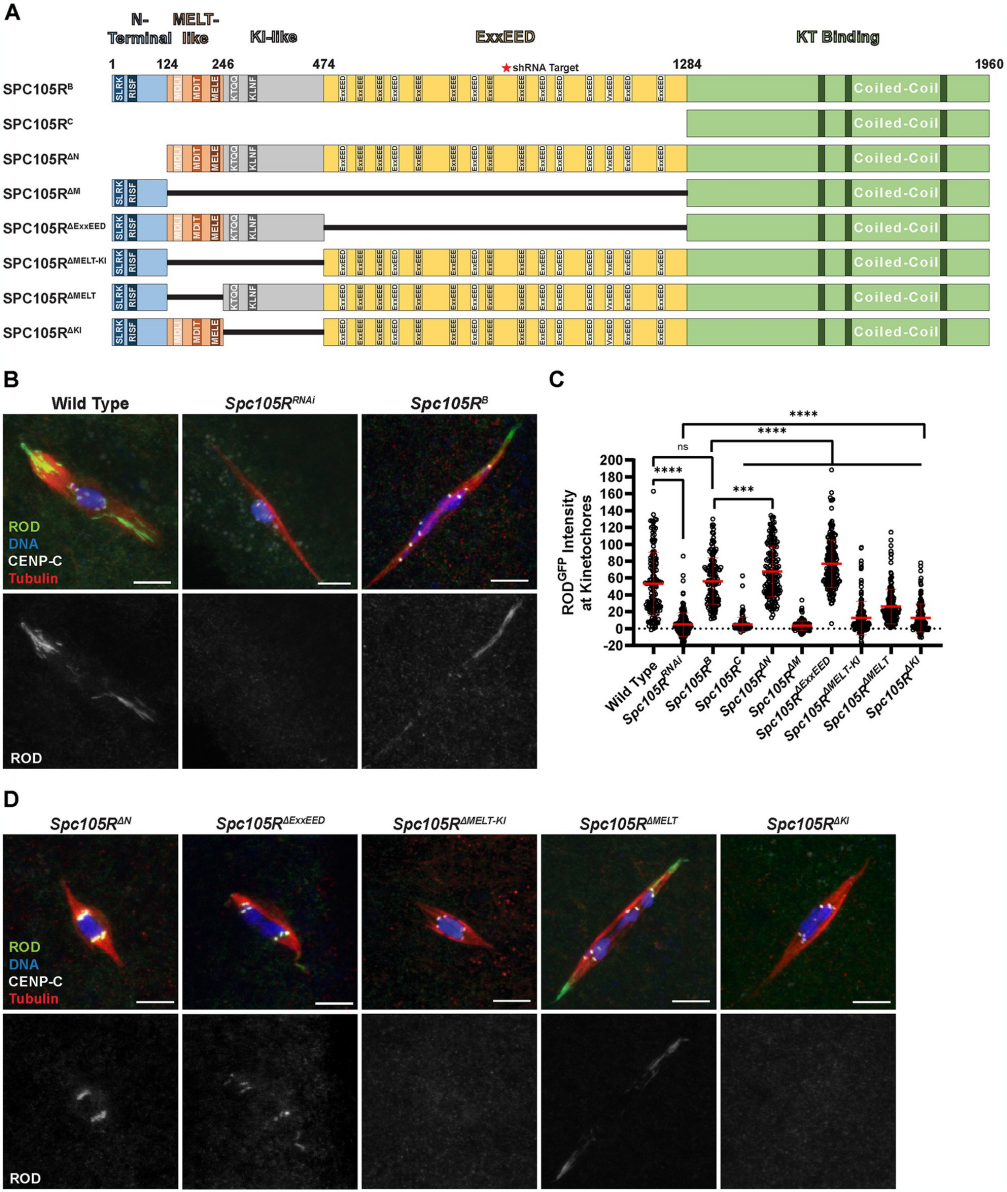
ROD localization depends on the MELT-KI domain of SPC105R. (A) Schematic of SPC105R^B^ domains in *Drosophila melanogaster* and deletion mutants used in this study. The coordinates on the schematic represent the first amino acid of each domain. (B) ROD^GFP^ localization in control and *Spc105R*^*RNAi*^ oocytes, with ROD^GFP^ in green, DNA in blue, CENP-C in white, and tubulin in red. Single channel images (bottom) show ROD^GFP^. (C) Quantification of ROD^GFP^ intensity at kinetochores normalized to background GFP signal in indicated oocytes (from left to right, n = 142, 169, 144, 144, 167, 138, 206, 159, 173, and 146 kinetochores). Error bars show mean ± s.d.; ****P<0.0001, ***P = 0.0006, ns = no significance (unpaired two-tailed t test). All *Spc105R* mutants are in an *Spc105R*^*RNAi*^ background targeting the endogenous *Spc105R*. (D) ROD^GFP^ localization in the indicated *Spc105R* mutant oocytes, with ROD^GFP^ in green, DNA in blue, CENP-C in white, and tubulin in red. Single channel images (bottom) show ROD^GFP^. All images are maximum intensity projections of z stacks. Scale bars represent 5 μm.

We also examined localization of INCENP and MAD1 in *Spc105R* deletion mutants to investigate the domains of SPC105R required for SAC protein localization (Fig 3A). Because *Spc105R* is an essential gene, we used a combination of *Spc105R*^*RNAi*^ and RNAi-resistant transgenes to examine mutants in oocytes. The control for each mutant was the RNAi-resistant wild-type transgene, *Spc105R*^*B*^. MAD1 kinetochore localization was reduced in mutants such as *Spc105R*^*C*^ or *Spc105R*^*ΔM*^ that deleted motifs (MELT, KI, and ExxEED) in the central region of SPC105R (S3A and S3B Fig). INCENP kinetochore localization was also lost in the majority of *Spc105R*^*C*^ or *Spc105R*^*ΔM*^ oocytes, but due to the diffuse chromatin localization, this was not reflected in significant differences in intensity (S3C and S3D Fig). These results suggest that the MELT-like, KI-like, and ExxEED repeats are involved in recruiting MAD1 and the CPC to the kinetochore.

MPS1 localization depends on SPC105R, as shown by its drastic reduction in *Spc105R*^*RNAi*^ oocytes (S4A and S4B Fig). *Ndc80*^*RNAi*^ oocytes also had a decrease in MPS1 localization, but this was less severe than *Spc105R*^*RNAi*^ oocytes, indicating that the majority of MPS1 is recruited either directly or indirectly by SPC105R (S4A and S4C Fig). Similarly, the *Spc105R*^*C*^, *Spc105R*^*ΔM*^, and *Spc105R*^*ΔExxEED*^ mutants had significant decreases in MPS1 localization, but not to the same degree as the *Spc105R*^*RNAi*^ oocytes (S4B and S4D Fig). Each of these mutants retains the C-terminal domain of SPC105R that recruits NDC80 [22]. Thus, these mutants may recruit more MPS1 than *Spc105R*^*RNAi*^ oocytes because NDC80 recruits some MPS1. Therefore, these results suggest that MPS1 localization depends on both SPC105R and NDC80.

ROD localization also depends on SPC105R, as shown by its reduction in *Spc105R*^*RNAi*^ oocytes (Fig 3B and 3C). In comparison to the other SAC proteins, recruitment of RZZ depends on a smaller and specific domain of SPC105R (Fig 3D). Oocytes with a deletion of everything except the KT binding domain (*Spc105R*^*C*^) or a deletion of all the sequences between the N-terminal and C-terminal domains (MELT-like, KI-like, and ExxEED, termed *Spc105R*^*ΔM*^) had almost no ROD localization, similar to *Spc105R*^*RNAi*^ oocytes. Furthermore, a deletion of the MELT-KI (*Spc105R*^*ΔMELT-KI*^), but not the ExxEED domain, had reduced ROD localization. Smaller deletions of only the KI and MELT domains (*Spc105R*^*ΔKI*^ and *Spc105R*^*ΔMELT*^) resulted in significantly reduced ROD localization at the kinetochores, although these intensities were higher than in the *Spc105R*^*ΔMELT-KI*^ deletion. These results suggest that ROD is recruited by both the MELT and KI regions.

**Fig 4:**
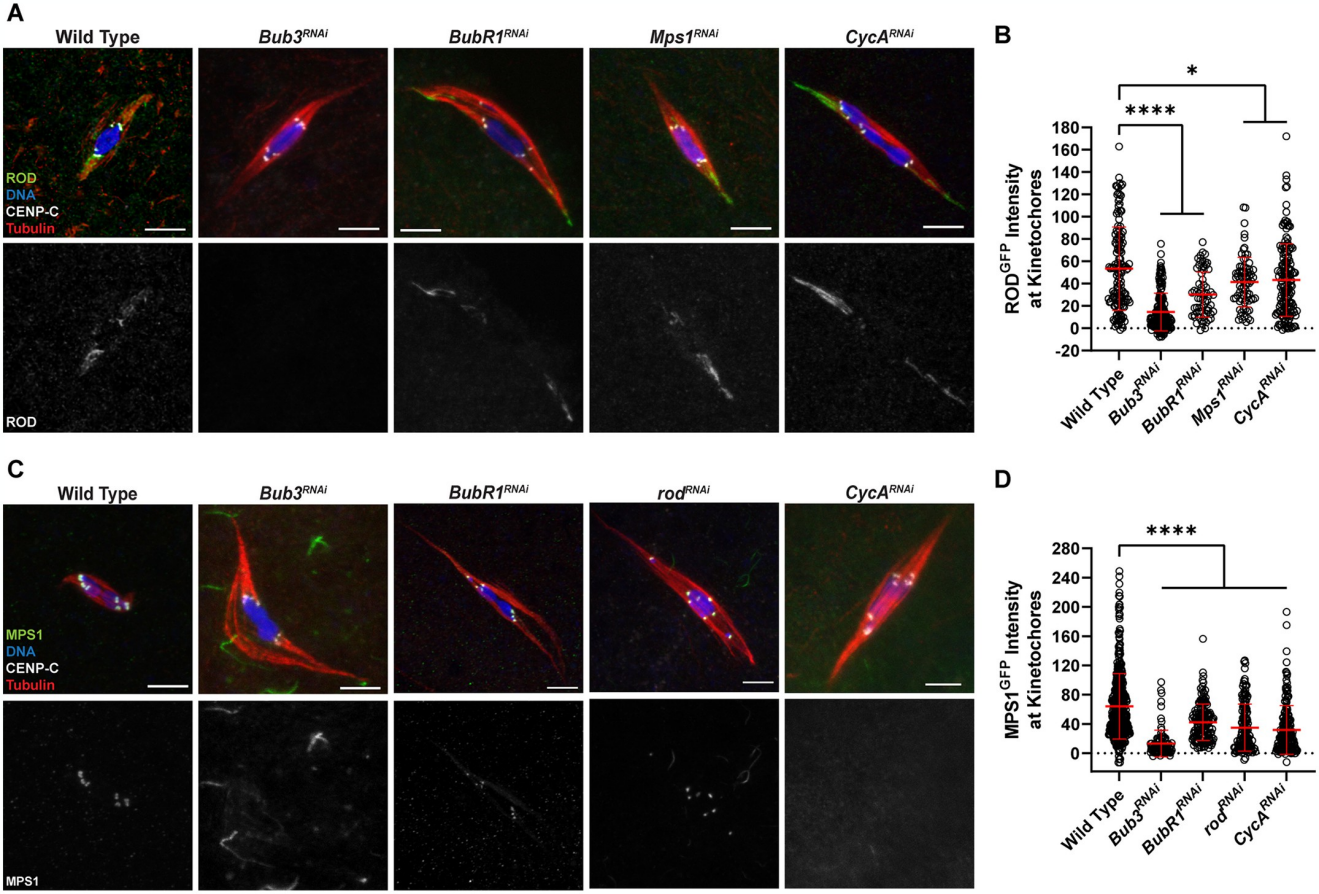
ROD and MPS1 localization depend on SAC genes. (A) ROD^GFP^ localization in wild-type, *Bub3*^*RNAi*^, *BubR1*^*RNAi*^, *Mps1*^*RNAi*^, and *CycA*^*RNAi*^ oocytes with ROD^GFP^ in green, DNA in blue, CENP-C in white, and tubulin in red. Single channel images (bottom) show ROD^GFP^. (B) Quantification of ROD^GFP^ intensity at kinetochores, normalized to background GFP signal in indicated oocytes (from left to right, n = 142, 170, 66, 76, and 155 kinetochores). Error bars show mean ± s.d.; ****P<0.0001, *P≤0.0123 (unpaired two-tailed t test). (C) MPS1^GFP^ localization in wild-type, *Bub3*^*RNAi*^, *BubR1*^*RNAi*^, *rod*^*RNAi*^, and *CycA*^*RNAi*^ oocytes with MPS1^GFP^ in green, DNA in blue, CENP-C in white, and tubulin in red. Single channel images (bottom) show MPS1^GFP^. (D) Quantification of MPS1^GFP^ intensity at kinetochores, normalized to background GFP signal in indicated oocytes (from left to right, n = 455, 97, 149, 180, and 152 kinetochores). Error bars show mean ± s.d.; ****P<0.0001 (unpaired two-tailed t test). All images are maximum intensity projections of z stacks. Scale bars represent 5 μm.

We also tested if the localization of ROD and MPS1 depended on other SAC proteins. ROD (Fig 4A and 4B) and MPS1 (Fig 4C and 4D) localization was significantly reduced in both *Bub3*^*RNAi*^ and *BubR1*^*RNAi*^ oocytes. The more significant reductions that were observed with *Bub3* knockdown could be due to less efficient *BubR1*^*RNAi*^ (see Methods), or redundancy between BUBR1 and BUB1. We also tested the effect of *Cyclin A* knockdown because it may regulate MPS1 localization [38]. *CycA*^*RNAi*^ oocytes had a significant reduction in MPS1 localization, although not as strong as *Bub3*^*RNAi*^, and a less significant reduction in ROD localization. Furthermore, MPS1 and ROD localizations were inter-dependent, although there was a milder reduction of ROD in *Mps1*^*RNAi*^. These results suggest ROD and MPS1 localizations depend on both BUB3 and Cyclin A.

### RZZ and MPS1, but not other SAC proteins, are required for meiosis I biorientation

**Fig 5:**
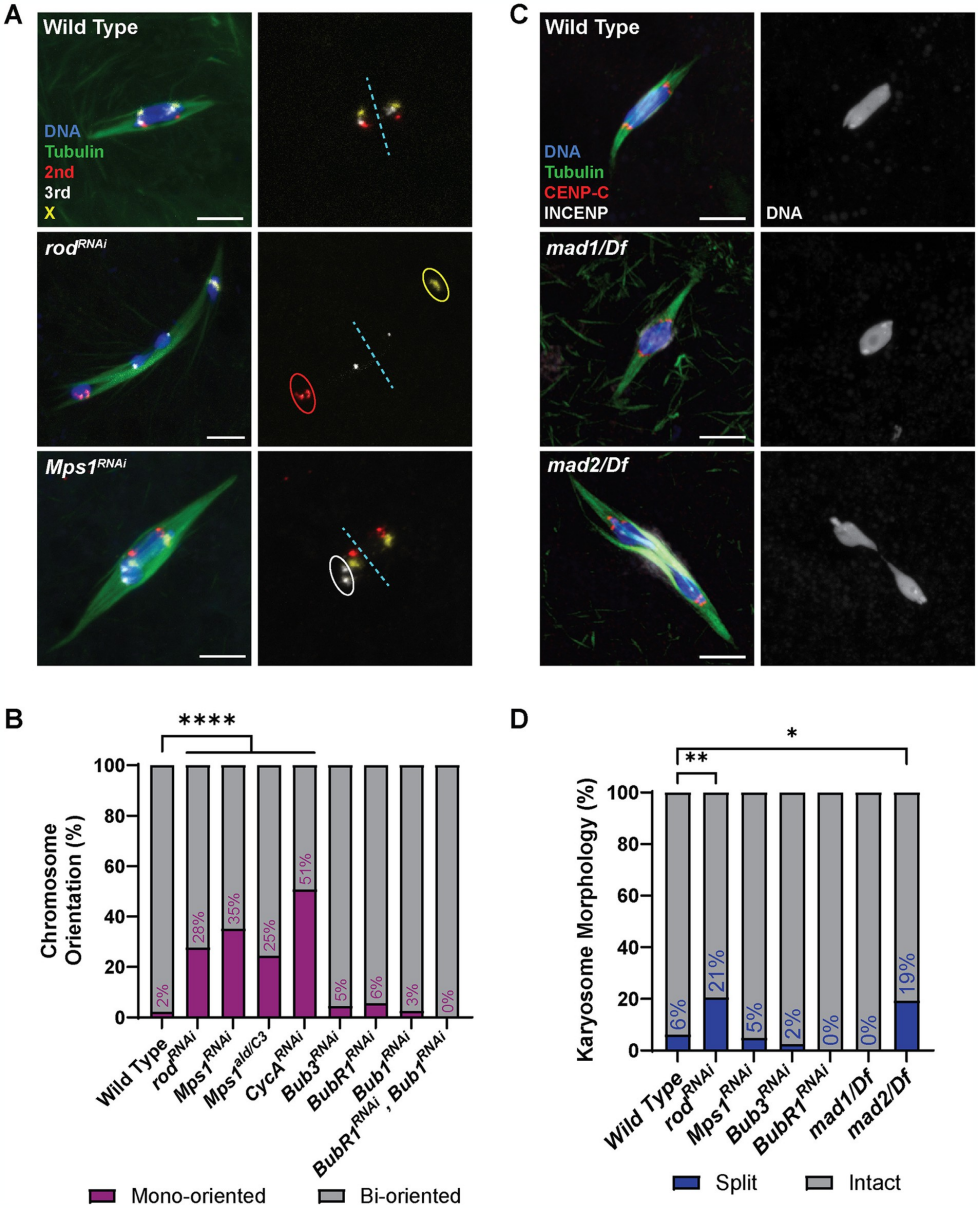
Meiotic chromosome alignment in SAC RNAi oocytes. (A) Representative images depicting chromosome orientation in wild-type, *rod*^*RNAi*^, and *Mps1*^*RNAi*^ oocytes. Images show DNA (blue), tubulin (green), the X chromosome (yellow), the second chromosome (red), and the third chromosome (white). FISH probes are also shown in a separate channel, with examples of mono-oriented chromosomes circled. The dashed blue line splits the karyosome in half to indicate which probes are mono-oriented. All images are maximum intensity projections of z stacks. Scale bars represent 5 μm. (B) Percent of chromosomes per oocyte with the indicated orientation (from left to right, n = 132, 65, 114, 94, 65, 66, 87, 39, and 30 pairs of chromosomes). ****P<0.0001 (Fisher’s exact test). (C) Examples of wild-type, *mad1*^*1*^, and *mad2*^*EY21687*^ mutant oocytes, with tubulin in green, DNA in blue, CENP-C in red, and INCENP in white. Single channel images show DNA. (D) Percent of oocytes with the specified karyosome morphology (from left to right, n = 114, 102, 103, 41, 31, 32, and 31 oocytes). **P = 0.0020, *P = 0.0375 (Fisher’s exact test).

To determine which SAC genes are required for biorientation during meiosis I, we used fluorescent in-situ hybridization (FISH) probes against the pericentromeric regions of the 2^nd^, 3^rd^, and X chromosomes. Biorientation was defined as two homologous centromeres at opposite ends of the karyosome. A mono-orientation defect was defined as the two homologous centromeres being in the same half of the karyosome (Figs 5A and S2B). Oocytes from females expressing *rod*^*RNAi*^ or *Mps1*^*RNAi*^ had increased chromosome mono-orientation (Fig 5B). The results with *Mps1* are consistent with previous studies showing nondisjunction using fertile allele combinations of *Mps1* [39,40]. We also tested *CycA*^*RNAi*^ oocytes, and consistent with a previous report [41], found an elevated level of biorientation defects (Fig 5B).

In contrast, *BubR1*^*RNAi*^, *Bub1*^*RNAi*^, *and Bub3*^*RNAi*^ oocytes had insignificant frequencies of mono-orientation (Fig 5B). Similarly, we have previously shown that flies with RNAi-mediated knock down of *Bub1* are fertile and do not have increased nondisjunction [33]. BUBR1 is required for sister chromatid cohesion protection in *Drosophila*, however, this may have not been detected by our FISH assay at metaphase I [22,42]. Mutants of *mad1* and *mad2* are viable and fertile [43,44], so we used genetic crosses to measure the frequency of nondisjunction (see Methods). Neither mutant had a significant increase in nondisjunction (Table 1). We also used a balancer chromosome to suppress crossing over on the X chromosome (*Bwinscy*), thus testing the role of *mad1* and *mad2* in an achiasmate system [45]. Only a mild increase in nondisjunction was observed. These results suggest that most of the SAC proteins are not required for biorientation of homologous chromosomes at metaphase I, while the RZZ complex and MPS1 may be involved in error correction in addition to their roles in the meiotic SAC.

**Table 1:** Nondisjunction in *mad* mutants.

Genotype	XX	XY	XXY	XO	% NDJ
*mad2* ^*EY21687*^ */Df*	3857	3558	6	17	0.6
*mad1* ^*1*^ */Df*	805	769	1	6	1.0
*Bwinscy/+; mad2* ^*EY21687*^ */Df*	357	263	4	12	4.9
*Bwinscy/+; mad1* ^*1*^ */mad1* ^*1*^	427	321	9	4	3.4
*Bwinscy/+; mad1* ^*1*^ */+*	588	375	2	4	1.2
*Bwinscy/+; mad2* ^*EY21687*^ */+*	509	301	2	2	1.0

*mad2* Df = *Df(3L)Exel6107* and *mad1* Df = *Df(2R)ED1791*

Stage 14 oocytes arrest in metaphase I [35,46]. If the metaphase I arrest depends on the SAC, we would predict that SAC mutants would precociously bypass this arrest and have increased oocytes with split karyosomes. However, this was not observed in most oocytes, suggesting the metaphase I arrest does not depend on SAC activity (Fig 5C and 5D). The most striking exceptions were *rod*^*RNAi*^ and the *mad2* mutant. The split karyosome phenotype was less frequent than in mutants lacking crossing over [22,35], suggesting these genes could have a minor role in the metaphase I arrest, or in the case of ROD, the precocious anaphase could result from bi-orientation or attachment defects (see below).

These results suggest MPS1 and ROD may have two distinct functions, one in the SAC and another for biorientation of homologous chromosomes during meiosis I. Given how little is known about RZZ in meiosis, we focused on understanding how ROD regulates attachments of kinetochores to microtubules, and if streaming is involved in this process.

### ROD streaming is independent of end-on attachments

**Fig 6:**
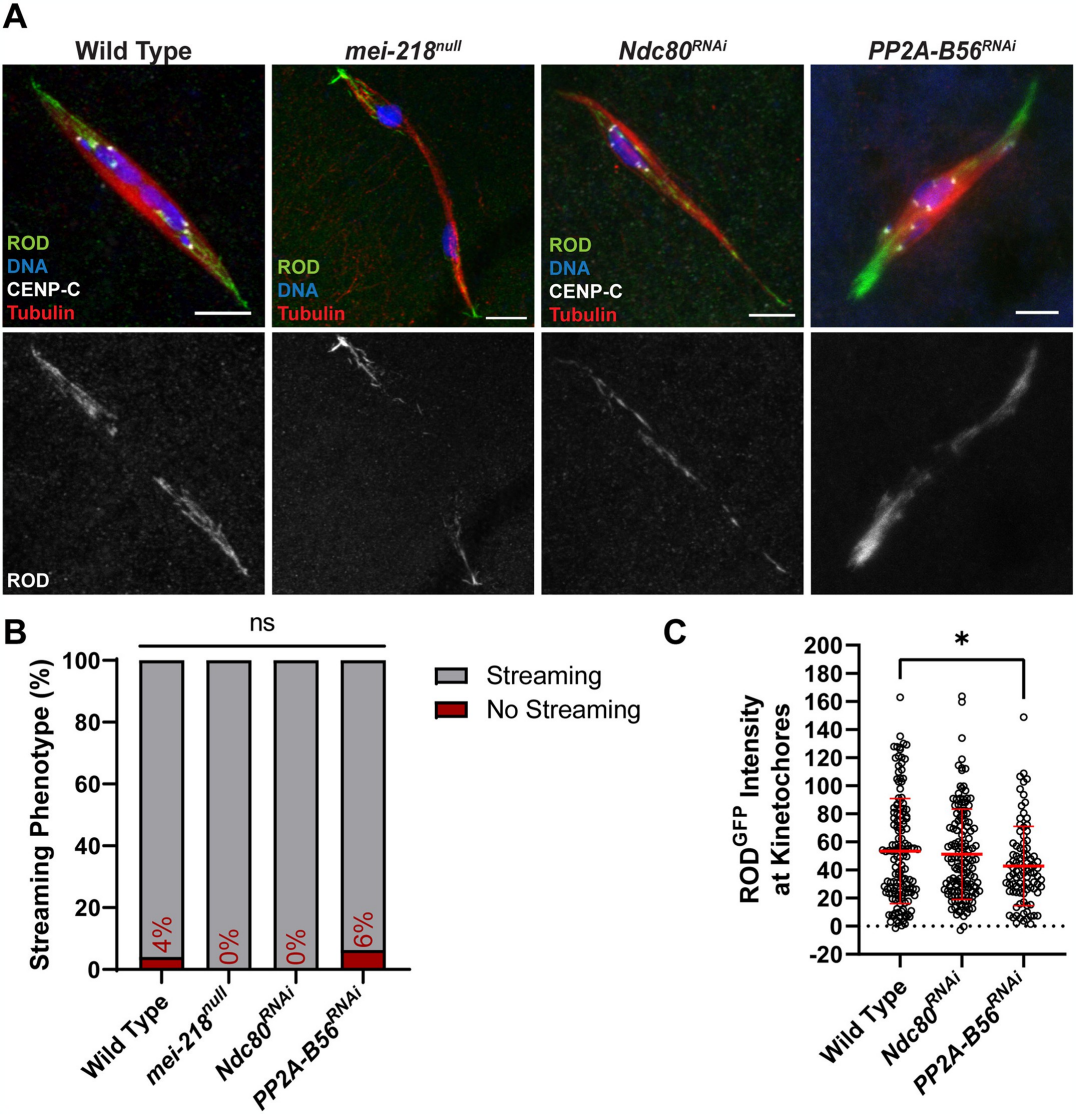
ROD streams in the absence of tension and end-on attachments. (A) ROD^GFP^ localization in wild-type, *mei-218*^*null*^, *Ndc80*^*RNAi*^, and *PP2A-B56*^*RNAi*^ oocytes with ROD^GFP^ in green, DNA in blue, CENP-C in white, and tubulin in red. Single channel images (bottom) show ROD^GFP^. All images are maximum intensity projections of z stacks. Scale bars represent 5 μm. (B) Percent of oocytes with ROD^GFP^ streaming in indicated genotypes (from left to right, n = 25, 15, 22, and 16 oocytes), ns = no significance (Fisher’s exact test). (C) Quantification of ROD^GFP^ intensity at kinetochores, normalized to background GFP signal in indicated oocytes (from left to right, n = 142, 139, and 92 kinetochores). Error bars show mean ± s.d.; *P = 0.0202 (unpaired two-tailed t test).

A striking feature of RZZ is its streaming off the kinetochores, but how streaming contributes to kinetochore attachments and biorientation in meiosis is not known. A small fraction of wild-type oocytes have no ROD streaming, which are likely oocytes in early prometaphase [27]. If streaming is in response to proper attachment of kinetochores to microtubules, we predicted that RZZ would not stream if errors were present or if end-on attachments could not form. To determine if biorientation errors prevented streaming, we examined ROD in *mei-218*^*null*^ oocytes, in which 90% of crossovers are absent, resulting in frequent chromosome segregation errors [47]. In the absence of crossovers, we still observed precocious anaphase and ROD streaming (Fig 6A and 6B). Thus, streaming occurs even in the absence of tension and in the presence of attachment errors.

To determine if attachment status regulates streaming, we examined ROD in *Ndc80*^*RNAi*^ oocytes, which lack end-on attachments [21] (S2A Fig). Despite the lack of end-on attachments, ROD streamed in all *Ndc80*^*RNAi*^ oocytes, and there was no effect on ROD accumulation at the kinetochores (Fig 6A–6C). Furthermore, oocytes lacking protein phosphatase 2A-B56 (PP2A-B56), which is also required for the creation of end-on attachments [48], still exhibited ROD streaming (Fig 6A–6C). Indeed, there may even be a reduction of ROD at the kinetochore in *PP2A-B56*^*RNAi*^ oocytes. These results demonstrate that end-on attachments are not required for the initiation of RZZ streaming.

### ROD is not required for lateral or end-on attachments

**Fig 7:**
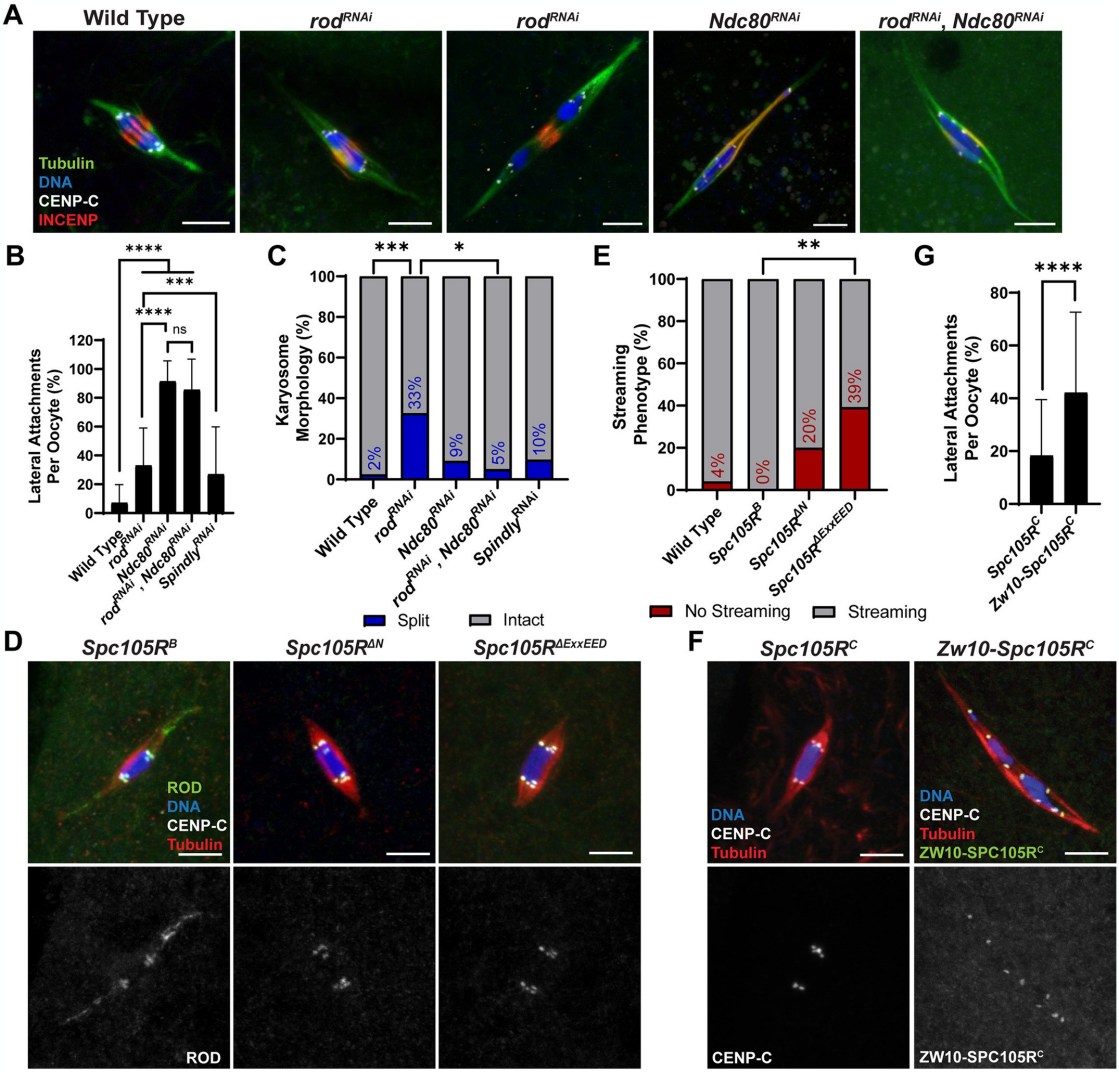
ROD is not required for microtubule attachments. (A) Wild-type; *rod*^*RNAi*^; *Ndc80*^*RNAi*^; and *rod*^*RNAi*^, *Ndc80*^*RNAi*^ oocytes with tubulin in green, DNA in blue, CENP-C in white, and INCENP in red. *Rod*^*RNAi*^ oocyte on the left has an intact karyosome and on the right a split karyosome. (B) Percent of kinetochores per oocyte with lateral kinetochore-microtubule attachments in indicated genotypes (from left to right, n = 40, 46, 30, 20, and 31 oocytes). Error bars show s.d.; ****P<0.0001, ***P = 0.0007, ns = no significance (unpaired two-tailed t test). (C) Percent of oocytes with the specified karyosome morphology in the indicated genotypes (from left to right, n = 41, 46, 22, 20, and 31 oocytes). ***P = 0.0002, *P = 0.0260 (Fisher’s exact test). (D) ROD^GFP^ localization in *Spc105R*^*B*^, *Spc105R*^*ΔN*^, and *Spc105R*^*ΔExxEED*^ oocytes with ROD^GFP^ in green, DNA in blue, CENP-C in white, and tubulin in red. Single channel images (bottom) show ROD^GFP^. All *Spc105R* mutants are in an *Spc105R*^*RNAi*^ background targeting the endogenous *Spc105R*. (E) Percent of oocytes with ROD^GFP^ streaming in indicated genotypes (from left to right, n = 25, 20, 20, and 28 oocytes); **P = 0.0012 (Fisher’s exact test). (F) *Spc105R*^*C*^ and *Zw10-Spc105R*^*C*^ oocytes (both in an *Spc105R*^*RNAi*^ background) with DNA in blue, CENP-C in white, tubulin in red, and ZW10-SPC105R^C^ in green (right). Single channel images (bottom) show CENP-C (left) or ZW10-SPC105R^C^ (right). (G) Percent of kinetochores per oocyte with lateral kinetochore-microtubule attachments in indicated genotypes (n = 42 and 51 oocytes). Error bars show s.d.; ****P<0.0001 (unpaired two-tailed t test). All *Spc105R*^*C*^ and *Zw10-Spc105R*^*C*^ oocytes are in an *Spc105R*^*RNAi*^ background targeting the endogenous *Spc105R*. All images are maximum intensity projections of z stacks. Scale bars represent 5 μm.

Microtubule nucleation from the kinetochore could depend on RZZ [49]. Thus, the biorientation defects observed in the *rod*^*RNAi*^ oocytes could be caused by the inability to create lateral or end-on attachments, or the creation of inaccurate attachments. If ROD is necessary for making end-on attachments, we would expect that oocytes depleted of *rod* alone would predominantly have lateral attachments. Although there was an increase in lateral attachments in *rod*^*RNAi*^ compared to wild-type oocytes, most attachments in *rod*^*RNAi*^ oocytes were end-on (Fig 7A and 7B). The increase in lateral attachments could be explained if *rod*^*RNAi*^ oocytes are delayed in making end-on attachments, possibly due to frequent biorientation errors. To test whether ROD was necessary for forming lateral attachments, we knocked down both *rod* and *Ndc80* (*rod*^*RNAi*^, *Ndc80*^*RNAi*^). Similar to *Ndc80*^*RNAi*^, we observed that *rod*^*RNAi*^, *Ndc80*^*RNAi*^ oocytes had primarily lateral attachments, suggesting that ROD is not required for making lateral attachments (Fig 7A and 7B).

As noted above, *rod*^*RNAi*^ oocytes have an elevated frequency of separated karyosomes. This phenotype was not observed in *rod*^*RNAi*^, *Ndc80*^*RNAi*^ oocytes, suggesting that the formation of end-on attachments is required for the separated karyosome phenotype (Fig 7A and 7C).

### ROD localization to the kinetochore may delay, but does not prevent, end-on attachments

Because ROD is not required for making lateral or end-on attachments, we reasoned that it could be required for regulating the transition from lateral to end-on attachments. This would align with a model proposed in mitotic cells in which high levels of RZZ at the kinetochores inhibit NDC80 from prematurely establishing end-on attachments [23,24]. In this model, RZZ streaming allows for the establishment of end-on attachments. If this was true in meiosis, we would expect oocytes in which ROD was exclusively on kinetochores and not streaming to have lateral attachments. This ROD behavior was observed in two *Spc105R* mutants. A deletion of the ExxEED repeats (*Spc105R*^*ΔExxEED*^) or the N-terminal region (*Spc105R*^*ΔN*^) had higher ROD intensities at the kinetochores and less streaming than in *Spc105R*^*B*^ oocytes (Figs 3D and 7D and 7E). However, oocytes with ROD only on kinetochores still exhibited end-on attachments (Fig 7D). These results suggest that the N-terminal region and ExxEED repeats of SPC105R may regulate streaming, and that RZZ streaming is not required for the creation of end-on attachments in *Drosophila* oocytes.

To directly test whether end-on attachments are inhibited when the RZZ complex is localized to the kinetochore, we stably targeted RZZ to the kinetochore. Because the recruitment of the RZZ components are interdependent [50], we reasoned that fusing *Zw10* to the KT-binding C-terminal domain of *Spc105R* (Z*w10-Spc105R*^*C*^*)* (S5A Fig), would result in the entire RZZ complex being permanently targeted to the kinetochores. Indeed, the ZW10-SPC105R^C^ fusion protein, detected using the 3XHA epitope-tag at the C-terminal end, was exclusively targeted to the kinetochores (Fig 7F). When *Zw10-Spc105R*^*C*^ was combined with an shRNA targeting the endogenous *Spc105R* (*Spc105R*^*RNAi*^), loss of endogenous SPC105R was confirmed cytologically using an antibody against the first 400 amino acids of SPC105R, which is absent in the fusion construct (S5B Fig). In addition, ROD localized to the kinetochores and did not stream in Z*w10-Spc105R*^*C*^ oocytes, indicating that the RZZ complex was restricted to the kinetochores (S5C Fig). ROD^GFP^ did not stream even when there was endogenous SPC105R present at the kinetochores, indicating that the ZW10-SPC105R^C^ fusion is a dominant negative mutation by preventing endogenous RZZ streaming from kinetochores. *Zw10-Spc105R*^*C*^ oocytes exhibited an increase in lateral attachments compared to *Spc105R*^*C*^ oocytes (Fig 7G). However, the majority of attachments were still end-on (~60%), indicating that RZZ may delay, but does not prevent, end-on attachments. Therefore, in agreement with the two *Spc105R* mutants (Fig 7D), streaming of RZZ is not required to establish end-on attachments in *Drosophila* oocytes, and end-on attachments occur while RZZ is localized to the kinetochore.

### Spindly regulates ROD streaming

**Fig 8:**
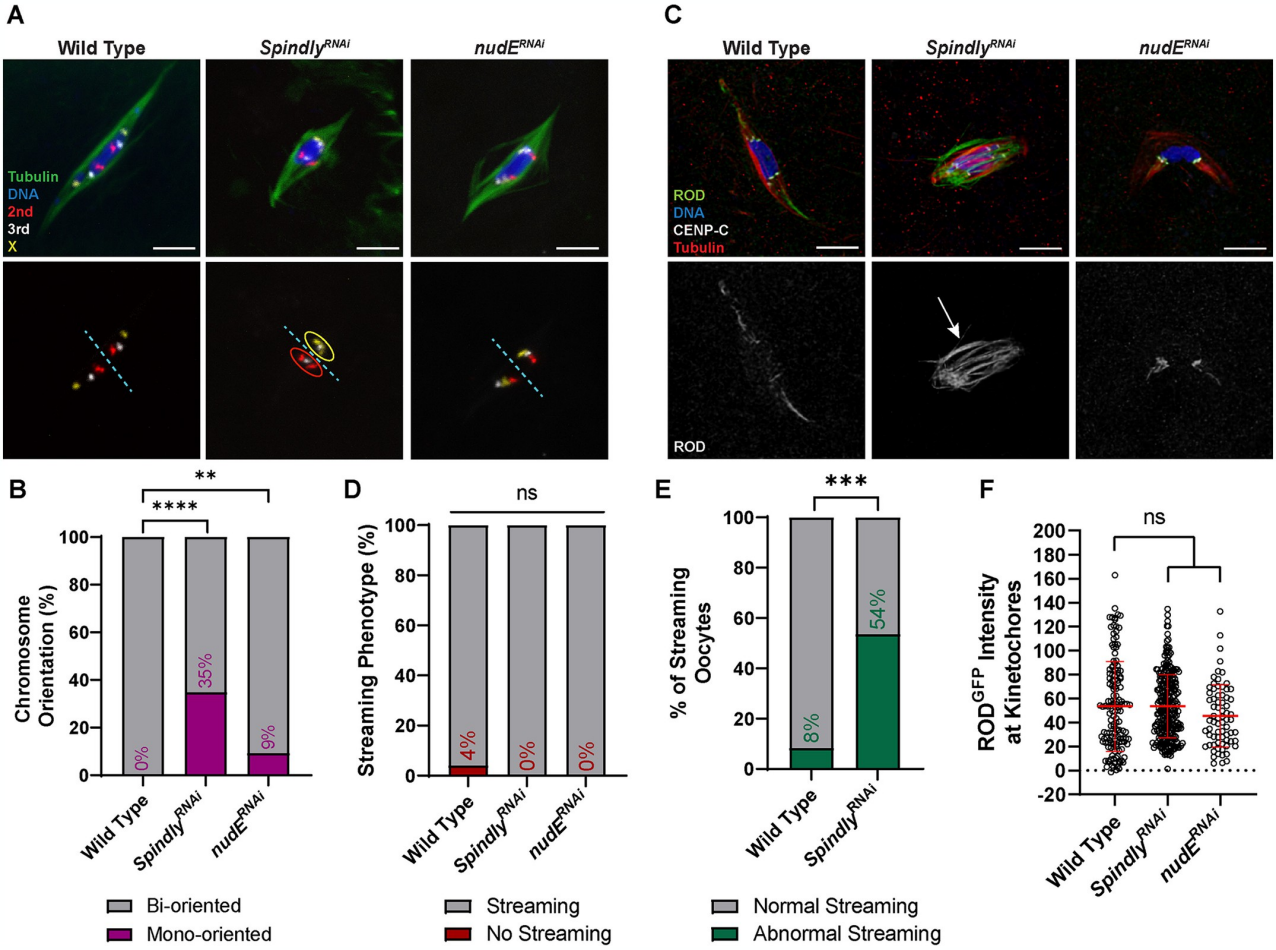
Requirement for Spindly in meiosis and interactions with ROD. (A) Representative images depicting chromosome orientation in wild-type, *Spindly*^*RNAi*^, and *nudE*^*RNAi*^ oocytes. Images show DNA (blue), tubulin (green), the X chromosome (yellow), the second chromosome (red), and the third chromosome (white). FISH probes are also shown in a separate channel, with examples of mono-oriented chromosomes circled. The dashed blue line splits the karyosome in half to indicate which probes are mono-oriented. (B) Percent of chromosomes per oocyte with the indicated orientation (from left to right, n = 86, 66, and 54 pairs of chromosomes); ****P<0.0001, **P = 0.0076 (Fisher’s exact test). (C) ROD^GFP^ localization in wild-type, *Spindly*^*RNAi*^, and *nudE*^*RNAi*^ oocytes with ROD^GFP^ in green, DNA in blue, CENP-C in white, and tubulin in red. Single channel images (bottom) show ROD^GFP^. Arrow points to abnormal streaming in the *Spindly*^*RNAi*^ oocyte. See also S6A Fig. (D) Percent of oocytes with ROD^GFP^ streaming in indicated genotypes (from left to right, n = 25, 41, and 10 oocytes); ns = no significance (Fisher’s exact test). (E) Percent of oocytes with abnormal streaming in wild-type or *Spindly*^*RNAi*^ oocytes (n = 24 and 43 oocytes); ***P = 0.0002 (Fisher’s exact test). Abnormal streaming is defined as having ROD^GFP^ in the central region between the centromeres. (F) Quantification of ROD^GFP^ intensity at kinetochores, normalized to background GFP signal in indicated oocytes (from left to right, n = 142, 256, and 72 kinetochores. Error bars show mean ± s.d.; ns = no significance (unpaired two-tailed t test). All images are maximum intensity projections of z stacks. Scale bars represent 5 μm.

Streaming is proposed to be dependent on the microtubule motor protein Dynein [51]. To further examine the role of RZZ streaming and its dependence on Dynein in meiosis, we investigated two Dynein-associated proteins previously implicated in RZZ removal from the *Drosophila* kinetochores: Spindly [52], and NudE [53]. We performed FISH on stage 14 oocytes to determine whether these two genes are required for chromosome biorientation (Fig 8A and 8B). There was only a slight increase in chromosome mono-orientation in *nudE*^*RNAi*^ oocytes compared to wild-type (9% and 0%, respectively). However, 35% of chromosomes were mono-oriented in *Spindly*^*RNAi*^ oocytes, suggesting that Spindly is necessary for accurate meiotic chromosome segregation.

To determine whether these proteins regulate streaming, we characterized ROD behavior in oocytes depleted of *Spindly* or *nudE* (Fig 8C–8E). Both *Spindly*^*RNAi*^ and *nudE*^*RNAi*^ oocytes exhibited ROD streaming (Fig 8D). This was surprising for Spindly, which has been shown to be required for streaming in *Drosophila* S2 cells [52]. However, *Spindly*^*RNAi*^ oocytes also exhibited unusual behavior, notably ROD streaming in the central spindle region between homologous centromeres in approximately 50% of oocytes (Figs 8C and 8E and S6A). Thus, Spindly may be required for proper streaming towards the spindle pole. To confirm that ROD was being efficiently removed and not accumulating at the kinetochores, we measured the intensity of ROD in *Spindly*^*RNAi*^ and *nudE*^*RNAi*^ oocytes (Fig 8F). Both RNAi oocytes had ROD levels similar to those in wild-type oocytes, indicating that ROD can still stream from kinetochores without NudE or Spindly. Therefore, while neither protein is required for RZZ streaming, Spindly may be required for RZZ to stream towards the spindle poles.

RZZ is proposed to recruit Spindly to the kinetochore, which is followed by streaming of both Spindly and RZZ [52,54]. To examine the localization of Spindly on the meiotic spindle, we used previously described *Spindly-GFP* fusion transgenes [55]. Oocytes expressing wild-type *Spindly-GFP* had weak localization throughout the spindle (S6B Fig). We also examined *Spindly* mutants with deletions of domains implicated in streaming: the N-terminus (a deletion of amino acids 1–228) and the Spindly Box (a deletion of amino acids 229–250). Both mutants had a dominant sterility phenotype when expressed in oocytes using *matα*, suggesting that these mutants interfere with the activity of the wild-type Spindly protein. In contrast to the wild-type oocytes, which had weak localization throughout the spindle, both mutants had pronounced localization near the centromeres. Spindly^ΔN^ localized to the kinetochores and spindle, while Spindly^ΔSB^ primarily localized to the kinetochores (S6B Fig). These results suggest that the Spindly Box domain is important for removal of Spindly from the kinetochores. The robust localization of these mutants was then used to test if ROD is required for Spindly localization, as shown in mitotic cells [52]. Indeed, Spindly^ΔSB^ was absent in *rod*^*RNAi*^ oocytes, indicating ROD is required for Spindly localization in oocyte meiosis (S6C and S6D Fig).

## Discussion

### The function of the SAC in oocytes

We analyzed the localization and meiotic phenotypes of several SAC proteins in *Drosophila* oocytes. Increased localization of MAD1, BUBR1, INCENP, and ROD at the kinetochores upon colchicine treatment indicates that *Drosophila* oocytes respond to loss of microtubules. However, increased localization of these proteins was not observed following loss of stable microtubule attachments to the kinetochores, the lack of tension, or the presence of biorientation errors. We propose that *Drosophila* oocytes have a modified “meiotic SAC” that is specific to promoting spindle assembly, rather than microtubule attachments to kinetochores [29].

Only two of the SAC proteins we tested were required for biorientation of homologous chromosomes at meiosis I, MPS1 [39,40] and ROD, suggesting that the SAC is not required for error correction. Similarly, *C*. *elegans* may correct attachment errors using a mechanism that does not rely on the SAC [56]. The SAC may have a more prominent role in chromosome segregation in mouse oocytes that arrest in meiosis II, where increased aneuploidy has been observed with loss of SAC proteins [57,58]. However, the evidence comes from a variety of assays that may not be comparable to our FISH experiments, and one study concluded that Mad2 is dispensable for accurate meiotic chromosome segregation except in response to stress [59].

In most cases, the loss of SAC proteins in *Drosophila* oocytes does not result in progression into anaphase (Fig 5) or a rise in APC activity [60]. These observations suggest the meiotic SAC may not regulate the APC in metaphase I-arrested oocytes. We cannot conclude, however, whether the meiotic SAC would prevent progression into anaphase after ovulation because our method of observing the SAC involved microtubule depolymerization. The metaphase I arrest in *Drosophila* oocytes may be maintained by a SAC-independent mechanism relying on chiasmata and cohesion, with progression into anaphase I depending on cohesion release. This would explain why *Drosophila* oocytes lacking chiasmata and tension bypass metaphase I arrest and progress into anaphase I [35,61].

Some observations suggest that the meiotic SAC is silenced at the end of prometaphase I in oocytes. This could allow for developmental regulation of the metaphase I arrest, with progression into anaphase occurring only after oocytes enter the oviduct. Developmentally regulated SAC silencing would explain why ROD does not stream in prometaphase I oocytes [27], but does stream in wild-type metaphase I-arrested oocytes, even in the absence of end-on attachments or the presence of multiple biorientation errors [22,48]. Similarly, MAD2 localizes to centromeres in early (stage 13) but not late (stage 14) stage oocytes [27], and the metaphase I spindle has some anaphase-like characteristics such as high concentrations of the CPC on the central spindle [33,62]. In budding yeast meiotic cells, SAC components are removed from kinetochores and the SAC is silenced in a PP1-dependent manner to prevent prolonged arrest, indicating this may be a conserved feature of meiotic cells [63]. Thus, it may be more advantageous for an organism to complete meiosis and produce a gamete than to lose the oocyte due to an extended SAC arrest.

### Recruitment of checkpoint proteins during meiosis

Our results are consistent with the known role of KNL1/SPC105R in the recruitment of checkpoint proteins to the kinetochore [1]. We previously showed that BUBR1 is recruited by both the MELT-KI and C-terminal regions of SPC105R [22]. MAD1, INCENP, and probably BUB3 depend on the MELT-KI and ExxEED repeat regions of SPC105R, which correspond to the repeated MELT motifs found in vertebrate KNL1 [32]. In the absence of SPC105R, recruitment of MAD1 shifts to the pericentromeric regions. Thus, there is a mechanism for SPC105R-independent recruitment of SAC proteins. Human cells may also have two pathways to recruit SAC proteins, one of which is active in the absence of KNL1 [64]. Thus, centromere components may recruit SAC proteins. In *Drosophila*, this could be facilitated by ZW10, which interacts with and is recruited by centromere protein CAL1 [65]. Alternatively, SAC activity may not require centromeres, because mitotic delays have been observed in the absence of CENP-A/CID in *Drosophila* [66].

### Multiple functions and recruitment factors for MPS1 and RZZ

We have shown that MPS1 and the RZZ complex have roles in error correction and in the SAC, while five SAC genes (*Mad1*, *mad2*, *BubR1*, *Bub1*, *and Bub3*) are not required for error correction. These results suggest that error correction is not under meiotic SAC control and involves a different mechanism. Furthermore, MPS1 and RZZ required for error correction and the meiotic SAC may be recruited to the kinetochore by different mechanisms. MPS1 was originally discovered with the mutation *ald* as being required for meiotic chromosome segregation [67], and subsequent studies have characterized its role in *Drosophila* meiosis [39,40] and mouse oocytes [68]. While MPS1 has been shown to be recruited by NDC80 [69–72], two observations show that a significant amount of MPS1 is also recruited independently of NDC80, possibly by SPC105R or SPC105R-interacting proteins. First, we observed a more severe decrease in MPS1 localization upon *Spc105R* depletion than *Ndc80* depletion. Second, we observed a decrease in MPS1 localization in *Spc105R* mutants that still recruit NDC80. While the NDC80-dependent MPS1 could regulate attachments [69,73], the NDC80-independent MPS1 could function in the meiotic SAC. However, additional studies will be needed to determine the contributions of NDC80 and SPC105R to MPS1 localization and function.

Like MPS1, our data suggest that there are two separate RZZ populations: one that is recruited by BUB3 and BUBR1 and is involved in the SAC and a second that is recruited by the MELT-KI domains of SPC105R and is important for error correction. The SAC population of ROD that depends on BUB3 could recruit other checkpoint proteins like MAD1 and MAD2 [74,75]. These conclusions are based on the observation that unlike *rod*^*RNAi*^, *Bub3*^*RNAi*^ and *BubR1*^*RNAi*^ oocytes do not have biorientation defects, suggesting that BUB3 and BUBR1 do not recruit the population of RZZ which is involved in error correction. Furthermore, there is a substantial amount of ROD^GFP^ remaining after *Bub3* and *BubR1* are knocked down, arguing that they do not recruit the total population of RZZ at kinetochores. However, the *Spc105R*^*ΔMELT-KI*^ mutant (deleting amino acids 154–840) abolishes nearly all ROD^GFP^ localization and does have biorientation defects [22]. Therefore, the MELT-KI domains of SPC105R are likely involved in recruiting the populations of RZZ involved in error correction, independently of BUB3.

The MELT-KI region of SPC105R is highly conserved [76]. Indeed, our results refine observations made in HeLa cells showing that the N-terminal domain of KNL1 recruits RZZ [77,78]. In *Drosophila* cell lines, a region within the KI-like domain (amino acids 266–384) and BUBR1 are required for RZZ recruitment to kinetochores [79]. Interestingly, this study also showed that the recruitment of RZZ by SPC105R could promote chromosome movement towards the spindle poles. Like this study, we found the KI domain recruits the majority of RZZ. It remains to be determined, however, why both the MELT and KI domains are required for RZZ recruitment and chromosome biorientation in meiosis I [22].

ROD and MPS1 localization was reduced in *CycA*^*RNAi*^ oocytes, and *CycA*^*RNAi*^ oocytes have biorientation defects in *Drosophila* oocytes [41]. Indeed, Cyclin A has been shown to regulate KT-MT attachments in mammalian cells [80,81], and in *Drosophila* mitotic cells, Cyclin A/CDK1 activates Polo kinase, which has recently been shown to regulate Spindly to prevent it from inhibiting RZZ [24]. Therefore, Cyclin A could be required for the functions of ROD and MPS1 associated with ensuring the accuracy of biorientation.

### The role of RZZ localization in attachment stabilization

Previous studies in *C*. *elegans*, *Drosophila*, and human mitotic cells have suggested a two-part model for how RZZ regulates KT-MT attachments [23,24,82,83]. First, RZZ interacts with NDC80 to inhibit end-on attachments. Second, Spindly-dependent RZZ streaming alleviates RZZ inhibition to allow for the formation of end-on attachments. Three of our observations are consistent with this hypothesis. First, knockdown of *Spindly* resulted in a decrease in end-on and an increase in lateral attachments. This would be expected if RZZ inhibits end-on attachments and Spindly negatively regulates RZZ. Second, Z*w10* fused to the C terminus of *Spc105R* led to complete RZZ retention on the kinetochores and increased lateral attachments. Third, we observed an increase in karyosome separation in *rod*^*RNAi*^ oocytes, which could be due to precocious stabilization of end-on attachments [22,84]. These results suggest that the RZZ complex at the kinetochore has a role in preventing or delaying end-on attachments.

However, some of our results are not consistent with this model. Oocytes expressing *Zw10-Spc105R*^*C*^ had primarily end-on attachments. A similar observation was made with two mutants (*Spc105R*^*ΔExxEED*^ and *Spc105R*^*ΔN*^) that had reduced ROD streaming but nonetheless formed end-on attachments. Therefore, if KT-localized RZZ inhibits end-on attachments, this can be overridden in oocytes. Finally, ROD streaming was observed in the absence of end-on attachments. Thus, physical removal of RZZ (or streaming) may not be required for the formation of end-on microtubule attachments, and alternative mechanisms for regulating this population of RZZ may exist. For example, a conformational change in RZZ may affect its regulation of attachments [23]. Understanding the relationship between the localization of the RZZ complex and its effects on microtubule attachments is crucial for analyzing its role in promoting accurate chromosome segregation during female meiosis.

### The role of Spindly in meiotic RZZ streaming and homolog biorientation

Spindly has a conserved role as a Dynein adaptor [85], and we have shown that RZZ recruits Spindly to kinetochores in *Drosophila* oocytes. Spindly has been shown in multiple organisms to recruit Dynein, resulting in the removal of the RZZ complex from the kinetochores [24,54,82,86]. This activity has been used to explain why depletion of RZZ can suppress defects in mitotic cells depleted of Spindly [23,24,82]. However, the role of Spindly in meiosis has not been reported.

Spindly is required for biorientation of homologous chromosomes during oocyte meiosis. The failure to remove RZZ from the kinetochores could be the reason for the meiotic biorientation defect. Contrary to the expectations from data in mitotic cells described above, however, knocking down *Spindly* did not abolish ROD streaming. Instead, we found that ROD displayed abnormal behaviors in oocytes depleted of *Spindly*, such as streaming in the opposite direction and localizing in the central spindle region between kinetochores. Therefore, it is likely that other proteins are involved in the removal of RZZ from the kinetochores in oocytes.

In the absence of Spindly in HeLa cells, alternative mechanisms are activated to remove kinetochore proteins [87]. One candidate is CENP-E, a plus-end directed motor required for expansion of the fibrous corona [88] and homolog biorientation in *Drosophila* oocytes [21]. Spindly, CENP-E, and RZZ might form a complex capable of bidirectional movement of the kinetochores [89]. In support of this hypothesis, RZZ is required for the poleward movement of cytoplasmic oligomers composed of a segment of SPC105R lacking the C-terminal kinetochore localization domain [90]. Our observation that RZZ streaming off the kinetochore is not required for end-on attachments suggests that an alternative model is needed to explain how RZZ regulates microtubule attachments. We suggest an important role for RZZ is the movement of kinetochores laterally along the sides of microtubules. This could involve moving towards a pole (minus-end directed) or changing poles (plus-end directed). Understanding how RZZ regulates biorientation may depend on reconciling the relative importance of two activities: RZZ moving kinetochores with motors like Dynein and CENP-E and RZZ blocking or delaying end-on attachments.

## Materials and methods

### *Drosophila* Genotypes and Expression of Transgenes and shRNAs

The transgenes and mutants used in this study are listed in Table 2. The localization of SAC proteins was examined in *Drosophila* oocytes using green fluorescent protein (GFP) fusion transgenes, most under the control of their own promoter. To examine MPS1, we used a construct used extensively in cell culture but modified for making transgenics (*P{EGFP-Mps1*.*F}* = *Mps1*^*GFP*^) [91,92]. To visualize the RZZ complex in oocytes, we used a construct where ROD was tagged with GFP at the N-terminus and under the control of its own promoter (*P{gEGFP-rod}* = *rod*^*GFP*^) [25] (S1A Fig). Expression of either transgene resulted in very low levels (~0.25%) of X-chromosome nondisjunction (S2 Table), which was comparable to wild-type strains [93]. This is consistent with previous studies showing that this transgene can rescue the lethality of *rod* mutants [25,94].

**Table 2:** *Drosophila* genotypes used in this work.

Genotype	Abbreviation	Reference
*P{w*^*+mC*^ = *matalpha-GAL-VP16}V37*	*matα*	[100]
*P{Val22*:*rod-153}attP40*	*rod* ^*RNAi*^	This paper
*P{gEGFP-rod}*	*rod* ^*GFP*^	[25]
*P{EGFP-Mps1*.*F}*	*Mps1* ^*GFP*^	[91]
*P{Mad1-GFP}*	*Mad1* ^*GFP*^	[44]
*P{BubR1*.*GFP}*	*BubR1* ^*GFP*^	[74]
*P{gEGFP-Bub3}III*.*3*	*Bub3* ^*GFP*^	[101]
*P{w*^*+mC*^ = *UASp-Spc105RB}*	*Spc105R* ^*B*^	[22]
*P{UASp-Spindly*.*RR*.*GFP}*	*Spindly* ^*GFP*^	[55]
*P{w*^*+mC*^ = *UASp-Zw10-Spc105RC}*	*Zw10-Spc105R* ^*C*^	This paper
*P{TRiP*.*GL00392}attP2*	*Spc105R* ^*RNAi*^	[102]
*P{TRiP*.*GL00625}attP40*	*Ndc80* ^*RNAi*^	[102]
*P{TRiP*.*HMS01283}attP2*	*Spindly* ^*RNAi*^	[102]
*P{TRiP*.*HMS01868}attP40*	*nudE* ^*RNAi*^	[102]
*P{TRiP*.*GL00151}attP2*	*Bub1* ^*RNAi*^	[102]
*P{wrd* ^*GL00671*^ *wdb* ^*HMS01864*^ *}/TM3*	*PP2A-B56* ^*RNAi*^	[102]
*P{TRiP*.*GL00236}attP2*	*BubR1* ^*RNAi*^	[102]
*P{TRiP*.*HMS00789}attP2*	*Bub3* ^*RNAi*^	[102]
*Mad1* ^*1*^	*Mad1*	[44]
*Mad2* ^*EY21687*^	*mad2*	[43]
*mei-218* ^*6*^	*mei-218* ^*null*^	[103]
*Mps1* ^*ald*^	*Mps1* ^*ald*^	[67]
*Mps1* ^*C3*^	*Mps1* ^*C3*^	[39]

The UAS/GAL4 system was utilized to drive expression of many of the transgenes in oocytes. *P{w*^*+mC*^ = *matalpha-GAL-VP16}V37* (*Matα*) (Table 2) was used to induce expression expression of UASP transgenes and shRNAs, starting after DNA replication and the pachytene events of homolog pairing and meiotic recombination, and continuing throughout meiotic prophase until metaphase I [34].

A fusion between the C-terminal domain of *Spc105R* and the entire coding region of Z*w10* was generated by GenScript in pENTR4. The C-terminal fragment of SPC105R has been characterized previously and is sufficient for kinetochore localization [22]. The fusion gene was transferred into pPWH, which fused the coding region to a 3XHA tag at the C-terminal end. This was injected into embryos for germline transformation by Model Systems Injections.

### RNA Extraction and Quantification of RNAi Knockdown

Among three shRNA lines targeting *rod*, one (*P{Val22*:*rod-153}1attP40*, = *rod*^*RNAi)*^) was chosen because it had the strongest phenotype and most severe mRNA knockdown (4% of the wild-type). Expression of *rod*^*RNAi*^ using *matα* produced sterile females and ubiquitous expression caused lethality. To confirm the efficacy of the *rod*^*RNAi*^ in oocytes, we examined ROD^GFP^ in oocytes expressing the *rod* shRNA. The intensity of ROD^GFP^ at the kinetochores in *rod*^*RNAi*^ oocytes was reduced to nearly background levels (S1B and S1C Fig), indicating that the *rod* shRNA provides a strong knockdown of *rod*. Similarly, the intensity of MPS1^GFP^ at the kinetochores in *Mps1*^*RNAi*^ oocytes was significantly reduced (S1D and S1E Fig).

RT-qPCR was performed to determine knockdown of mRNAs for each shRNA. After the parental flies were crossed, mature female offspring with the correct genotype were collected into yeasted vials for three days. These flies were then ground in 1X phosphate-buffered saline (PBS) and filtered through meshes to isolate the stage 14 oocytes. 1 mL of TRIzol reagent was added and RNA was extracted per the manufacturer’s instructions (Thermo Fisher Scientific). Taqman RT-qPCR was performed to quantify RNAi knockdowns. 2 μg of isolated RNA was reverse transcribed into cDNA using the High Capacity cDNA Reverse Transcription Kit (Applied Biosystems). Afterwards, qPCR was performed using a TaqMan Assay (Thermo Scientific) with four replicates per reaction. The efficacy of RNAi knockdowns has been published for *Ndc80 GL00625* = 6% remaining, *Spc105R GL00392* = 13% remaining [21], *Bub1 GL00151* = 2% remaining [33], *BubR1 GLV21065* = 22% remaining, *BubR1 GL00236* = 27% remaining, and *Bub3 HMS00789* = 0.7% remaining.

### Measuring X-Chromosome Nondisjunction

To measure nondisjunction of the X chromosome, virgin females with the desired genotype were crossed to males with a dominant *Bar* mutation on the Y chromosome (*yw/B*^*S*^*Y*). This mutation led to a visible bar-eyed phenotype where the eye was thinner than wild-type eyes. The normal segregation of chromosomes would lead to female offspring (XX) with wild-type eyes and males (XY) with bar eyes. If a nondisjunction event occurred in the X chromosome, the following sex chromosome combinations were possible: XO, XXY, XXX, and YO. XO and XXY were viable and resulted in normal-eyed males and bar-eyed females, respectively. XXX and YO were lethal. To calculate nondisjunction rates while taking into account the lethal genotypes, the following equation was used: $\frac{\#\;flies\;with\;nondisjunction\;\times \;2}{\left(\#\;flies\;with\;nondisjunction\;\times \;2\right)\;+\;(\#\;of\;normal\;flies)}$. This rate determined whether a particular genotype resulted in increased meiotic nondisjunction.

### Cytology of Stage 14 Oocytes

Cytology of stage 14 oocytes was performed as previously described [95]. After the parental flies were crossed, mature female offspring with the correct genotypes were collected into yeasted vials for one to two days. These flies were then broken open in a blender while in 1x modified Robb’s buffer and filtered through meshes to isolate the stage 14 oocytes. If a colchicine treatment was needed, oocytes were incubated with 150 μM colchicine or 0.5% EtOH in modified Robb’s buffer for one hour to remove all microtubules. The oocytes were fixed with 5% formaldehyde and heptane before being rinsed in 1X PBS. The oocytes were then rolled between the frosted surface of a glass slide and a cover slip to remove the membranes and nutated for two hours in PBS/1% Triton X-100 to make them permeable to antibody staining. Afterwards, oocytes were washed in PBS/0.05% Triton X-100 and blocked for one hour in blocking buffer (0.5% BSA and 0.1% Tween-20 in 1X PBS). Subsequently, primary antibodies were added to the oocytes while the secondary antibodies were added to *Drosophila* embryos to remove any nonspecific binding. After nutating overnight at 4°C, the oocytes were washed in blocking buffer four times at room temperature for 15 minutes. The secondary antibodies were added and the oocytes were nutated for 3–4 hours at room temperature. The oocytes were then washed in blocking buffer with Hoechst33342 (10 μg/mL) to stain the DNA. The oocytes were washed twice more in blocking buffer and then were ready for mounting and imaging.

The primary antibodies used in this paper were tubulin monoclonal antibody DM1A at 1:50 conjugated to FITC (Sigma-Aldrich), tubulin E7 monoclonal antibody at 1:150 [96], rat anti-INCENP at 1:600 [97], guinea pig anti-CENP-C at 1:1000 [98], rat anti-tubulin at 1:300 (Millipore), rabbit anti-Spc105R (1:4000) [99], rabbit anti-GFP (1,200) (Thermo Fisher), rat anti-HA (Roche, 1:50). Multiply preabsorbed secondary antibodies were from Jackson ImmunoResearch, and were conjugated to Alexa 647, Cy3, Alexa 594, or Alexa 488.

To visualize pairs of homologous chromosomes, fluorescent *in situ* hybridization (FISH) was performed. After oocytes fixation, 2X SSC was added and the membranes were removed by rolling the oocytes between glass slides. The oocytes were then nutated in increasing concentrations of formamide solution (20%, 40%, and 50%) before being added to the hybridization solution and probes. The probes were synthesized by IDT, and recognize the 359 bp repeats on the X chromosome (labeled with AlexaFluor 594), the AACAC repeats on the second chromosome (labeled with Cy3), or the dodeca repeats on the third chromosome (labeled with Alexa 647). This was followed by incubation in 80°C for 20 minutes and then 37°C overnight. The next day, the oocytes were washed in 50% formamide solution twice at 37°C and 20% formamide once at room temperature. The oocytes were then rinsed three times in 2X SSCT, once in 2X PBST, and nutated in blocking buffer for 4 hours at room temperature. Afterwards, mouse anti-tubulin-FITC antibody (Sigma) was added (1:50) and the oocytes were nutated overnight at room temperature. The next day, the oocytes were washed in blocking buffer with Hoechst33342 (10 μg/mL). After washing twice more with blocking buffer, the oocytes were mounted and imaged.

### Imaging oocytes, image analysis, and statistical analysis

Oocytes were mounted on a glass slide in SlowFade Gold (Invitrogen) and images were taken with a 63x lens on a Leica SP8 or Stellaris microscope. Protein intensities were quantified using Imaris image analysis software (Bitplane). To measure the intensity of proteins at kinetochores, spots marking the kinetochores and random areas of the background were created. The average intensity of the protein of interest at the background was subtracted from the intensity at each kinetochore to generate the normalized intensity value. GraphPad Prism software was used to graph data and perform statistical analysis. All statistical tests and sample sizes are reported in the figure legends.

## Supporting information

S1 Fig Validation of *rod* and *mps1* reagents. (A) Wild-type oocyte with ROD^GFP^ in green, ZW10^HA^ in red, DNA in blue, and tubulin in white. Single channel images show ROD^GFP^ (middle) and ZW10^HA^ (right). (B) ROD^GFP^ localization in wild-type and *rod*^*RNAi*^ oocytes with ROD^GFP^ in green, DNA in blue, CENP-C in white, and tubulin in red. Single channel images (right) show ROD^GFP^. (C) Quantification of ROD^GFP^ intensity at kinetochores, normalized to background GFP signal in wild-type and *rod*^*RNAi*^ oocytes (n = 142 and 126 kinetochores). Error bars show mean ± s.d.; ****P<0.0001 (unpaired two-tailed t test). (D) MPS1^GFP^ localization in wild-type and *Mps1*^*RNAi*^ oocytes with MPS1^GFP^ in green, DNA in blue, CENP-C in white, and tubulin in red. Single channel images (right) show MPS1^GFP^. (E) Quantification of MPS1^GFP^ intensity at kinetochores, normalized to background GFP signal in wild-type and *Mps1*^*RNAi*^ oocytes (n = 75 and 71 kinetochores). Error bars show mean ± s.d.; ****P<0.0001 (unpaired two-tailed t test). All images are maximum intensity projections of z stacks. Scale bars represent 5 μm.
(TIF)

S2 Fig Schematic of chromosome orientation and kinetochore-microtubule attachment states. (A) Schematic example of an oocyte with end-on attachments, where the kinetochore (white) is associated with the end of the microtubule bundle (red), or an oocyte with lateral attachments, where the kinetochore is associated with the side of the microtubule bundle. (B) Schematic of an oocyte where each pair of homologous chromosomes is marked in a different color. A dashed line is shown splitting the karyosome in half, with homologous chromosomes in the same half of the karyosome being scored as mono-oriented (2^nd^ and X chromosomes) while homologs in different halves of the karyosome are scored as bi-oriented (3^rd^ chromosome).
(TIF)

S3 Fig MAD1 and INCENP localization depends on SPC105R. Control (*Spc105R*^*B*^) or mutant oocytes were incubated for one hour in 250 μM colchicine. (A) MAD1^GFP^ (green) localization in indicated genotypes, with DNA in blue, CENP-C in white, and tubulin in red. Single channel images (bottom) show MAD1^GFP^. (B) Quantification of MAD1^GFP^ intensity at kinetochores, normalized to background GFP signal (from left to right, n = 91, 256, 224, 174, 212, 208, and 236 kinetochores). Error bars show mean ± s.d.; ****P<0.0001, ***P = 0.0006 (unpaired two-tailed t test). (C) INCENP localization (green) in indicated genotypes with DNA in blue, CENP-C in white, and tubulin in red. Single channel images (bottom) show INCENP. (D) Quantification of INCENP presence at kinetochores (from left to right, n = 16, 29, 11, 31, 20, 18, 21, and 11 oocytes). Error bars show mean ± s.d.; ****P<0.0001, *P = 0.02 (Fisher’s exact test). All images are maximum intensity projections of z stacks. Scale bars represent 5 μm. All *Spc105R* mutants are in an *Spc105R*^*RNAi*^ background targeting the endogenous *Spc105R*.
(TIF)

S4 Fig MPS1 localization depends on SPC105R and NDC80. (A) MPS1^GFP^ localization in wild-type, *Spc105R*^*RNAi*^, and *Ndc80*^*RNAi*^ oocytes, with MPS1^GFP^ in green, DNA in blue, CENP-C in white, and tubulin in red. Single channel images (bottom) show MPS1^GFP^. (B) Quantification of MPS1^GFP^ intensity at kinetochores, normalized to background GFP signal in indicated oocytes (from left to right, n = 389, 195, 139, 160, 165, 198, 128, and 143 kinetochores). Error bars show mean ± s.d.; ****P<0.0001 (unpaired two-tailed t test). (C) Quantification of MPS1^GFP^ intensity at kinetochores, normalized to background GFP signal in wild-type and *Ndc80*^*RNAi*^ oocytes (n = 114 and 148 kinetochores). Error bars show mean ± s.d.; ****P<0.0001 (unpaired two-tailed t test). (D) MPS1^GFP^ localization in the indicated *Spc105R* mutants with MPS1^GFP^ in green, DNA in blue, CENP-C in white, and tubulin in red. All mutants are in an *Spc105R*^*RNAi*^ background targeting the endogenous *Spc105R*. Single channel images (bottom) show MPS1^GFP^. All images are maximum intensity projections of z stacks. Scale bars represent 5 μm.
(TIF)

S5 Fig ROD and SPC105R localization in Z*w10-Spc105R*^*C*^ oocytes. (A) Schematic of SPC105R^B^ and the ZW10-SPC105R^C^ fusion. (B) SPC105R localization in Z*w10-Spc105R*^*C*^, either in the presence or absence of *Spc105R*^*RNAi*^. SPC105R (green) was detected using an antibody which recognizes the N-terminal regions of SPC105R and does not detect SPC105R^C^. DNA is in blue, CENP-C in white, and tubulin in red. (C) ROD^GFP^ localization in oocytes expressing Z*w10-Spc105R*^*C*^, either in the presence or absence of *Spc105R*^*RNAi*^. ROD^GFP^ is in green, DNA in blue, CENP-C in white, and tubulin in red. All images are maximum intensity projections of z stacks. Scale bars represent 5 μm.
(TIF)

S6 Fig Localization of Spindly variants and dependence of Spindly on ROD. (A) Examples of normal (left) and abnormal (right) ROD^GFP^ streaming in *Spindly*^*RNAi*^ oocytes, with ROD^GFP^ in green, DNA in blue, CENP-C in white, and tubulin in red. Single channel images (bottom) show ROD^GFP^. Arrow points to abnormal streaming, which is defined as having ROD^GFP^ in the central region between the centromeres. (B) Spindly^GFP^ localization in wild-type or mutants of *Spindly*. Spindly^GFP^ is in green, DNA in blue, CENP-C in white, and tubulin in red. (C) Localization of Spindly^ΔSB.GFP^ in *rod*^*RNAi*^ oocytes, with Spindly^GFP^ in green, DNA in blue, CENP-C in white, and tubulin in red. Spindly^GFP^ and CENP-C are shown below the merged images. All images are maximum intensity projections of z stacks. Scale bars represent 5 μm. (D) Quantification of Spindly^GFP^ intensity at kinetochores in the indicated oocytes (from left to right, n = 123, 164, 83, and 75 kinetochores). Error bars show mean ± s.d.; ****P<0.0001 (unpaired two-tailed t test).
(TIF)

S1 Table Summary of SAC protein localization data. (DOCX)

S2 Table Meiotic X-chromosome nondisjunction in *rod* and *mps1* transgenes. (DOCX)

S1 Data Fluorescence imaging data and analysis. The file is organized into multiple tabs, with each tab containing the data from one figure.
(XLSX)
