# Supplementary figures and images for: Distinct checkpoint and homolog biorientation pathways regulate meiosis I in Drosophila oocytes
Source: PLoS Genet. 2025 Jan 29;21(1):e1011400. doi: 10.1371/journal.pgen.1011400 (PMC11809923; doi:10.1371/journal.pgen.1011400)

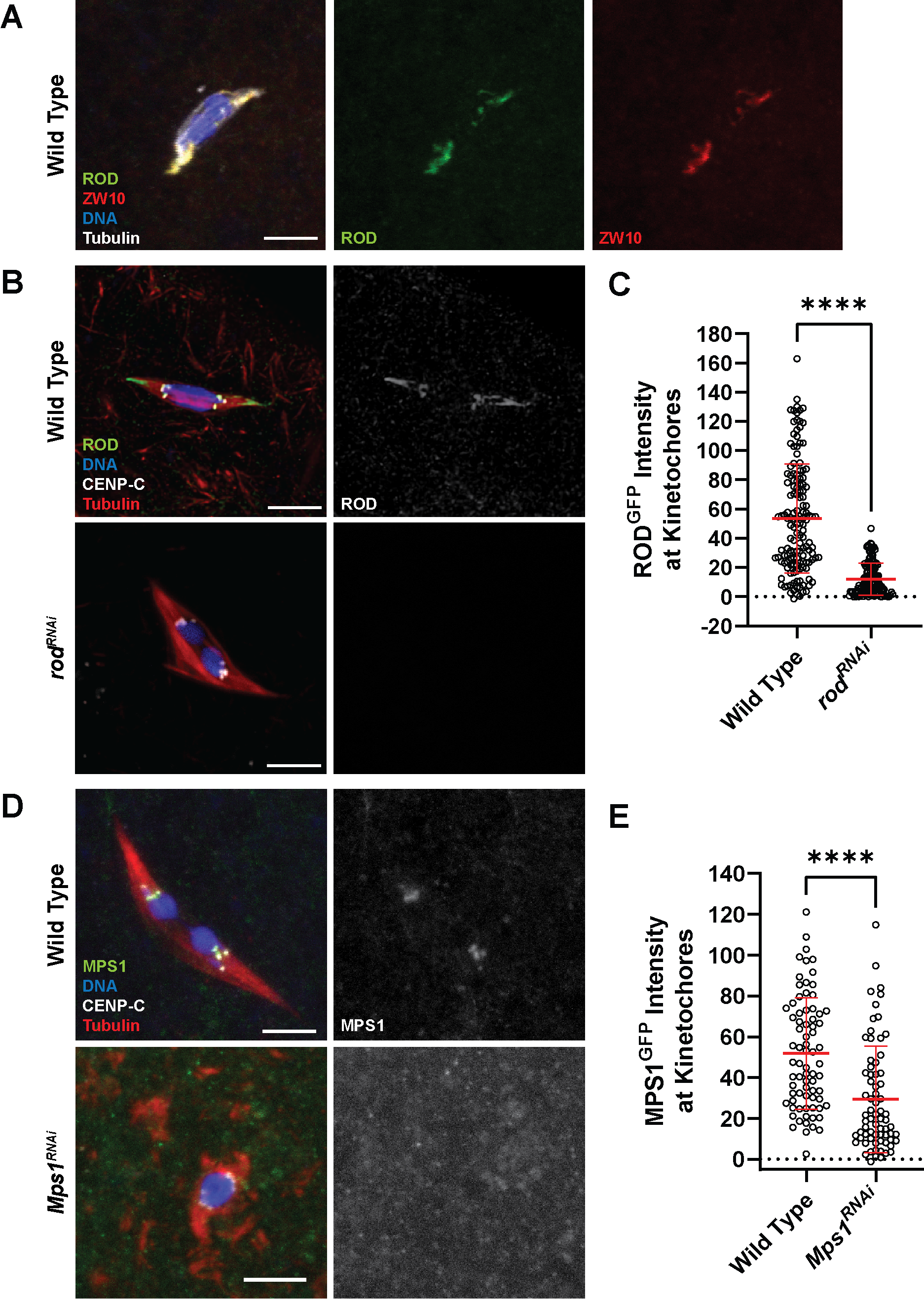

Supplement: S1 Fig — (A) Wild-type oocyte with RODGFP in green, ZW10HA in red, DNA in blue, and tubulin in white. Single channel images show RODGFP (middle) and ZW10HA (right). (B) RODGFP localization in wild-type and rodRNAi oocytes with RODGFP in green, DNA in blue, CENP-C in white, and tubulin in red. Single channel images (right) show RODGFP. (C) Quantification of RODGFP intensity at kinetochores, normalized to background GFP signal in wild-type and rodRNAi oocytes (n = 142 and 126 kinetochores). Error bars show mean ± s.d.; ****P<0.0001 (unpaired two-tailed t test). (D) MPS1GFP localization in wild-type and Mps1RNAi oocytes with MPS1GFP in green, DNA in blue, CENP-C in white, and tubulin in red. Single channel images (right) show MPS1GFP. (E) Quantification of MPS1GFP intensity at kinetochores, normalized to background GFP signal in wild-type and Mps1RNAi oocytes (n = 75 and 71 kinetochores). Error bars show mean ± s.d.; ****P<0.0001 (unpaired two-tailed t test). All images are maximum intensity projections of z stacks. Scale bars represent 5 μm. (TIF) [file pgen.1011400.s001.tif]

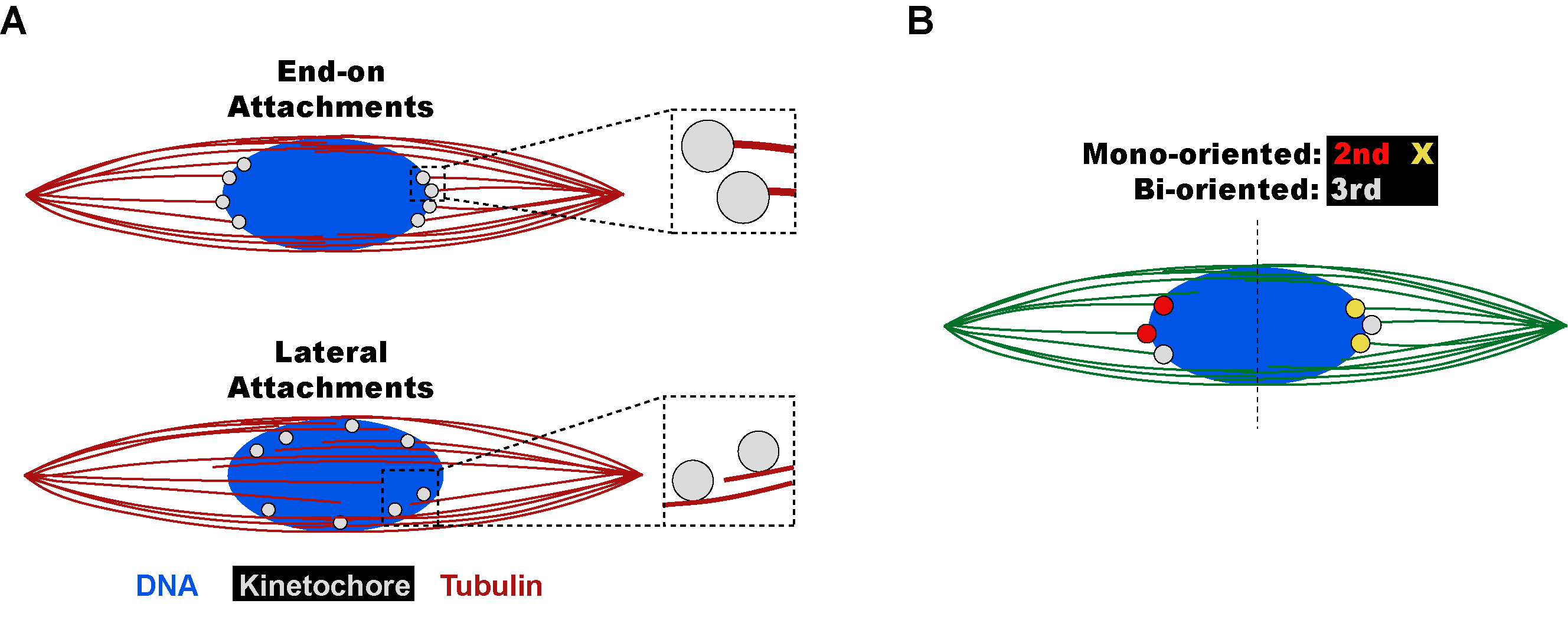

Supplement: S2 Fig — (A) Schematic example of an oocyte with end-on attachments, where the kinetochore (white) is associated with the end of the microtubule bundle (red), or an oocyte with lateral attachments, where the kinetochore is associated with the side of the microtubule bundle. (B) Schematic of an oocyte where each pair of homologous chromosomes is marked in a different color. A dashed line is shown splitting the karyosome in half, with homologous chromosomes in the same half of the karyosome being scored as mono-oriented (2nd and X chromosomes) while homologs in different halves of the karyosome are scored as bi-oriented (3rd chromosome). (TIF) [file pgen.1011400.s002.tif]

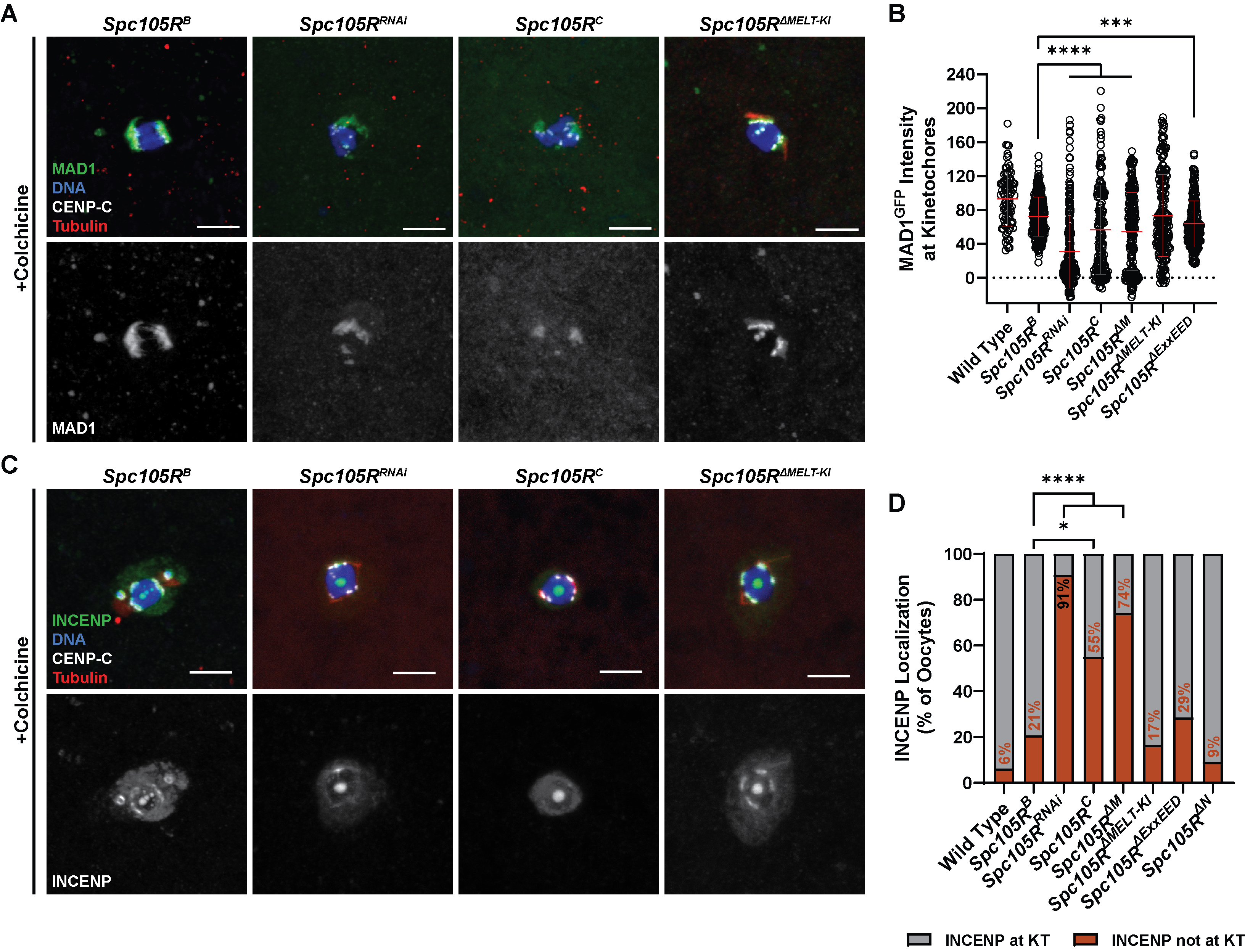

Supplement: S3 Fig — Control (Spc105RB) or mutant oocytes were incubated for one hour in 250 μM colchicine. (A) MAD1GFP (green) localization in indicated genotypes, with DNA in blue, CENP-C in white, and tubulin in red. Single channel images (bottom) show MAD1GFP. (B) Quantification of MAD1GFP intensity at kinetochores, normalized to background GFP signal (from left to right, n = 91, 256, 224, 174, 212, 208, and 236 kinetochores). Error bars show mean ± s.d.; ****P<0.0001, ***P = 0.0006 (unpaired two-tailed t test). (C) INCENP localization (green) in indicated genotypes with DNA in blue, CENP-C in white, and tubulin in red. Single channel images (bottom) show INCENP. (D) Quantification of INCENP presence at kinetochores (from left to right, n = 16, 29, 11, 31, 20, 18, 21, and 11 oocytes). Error bars show mean ± s.d.; ****P<0.0001, *P = 0.02 (Fisher’s exact test). All images are maximum intensity projections of z stacks. Scale bars represent 5 μm. All Spc105R mutants are in an Spc105RRNAi background targeting the endogenous Spc105R. (TIF) [file pgen.1011400.s003.tif]

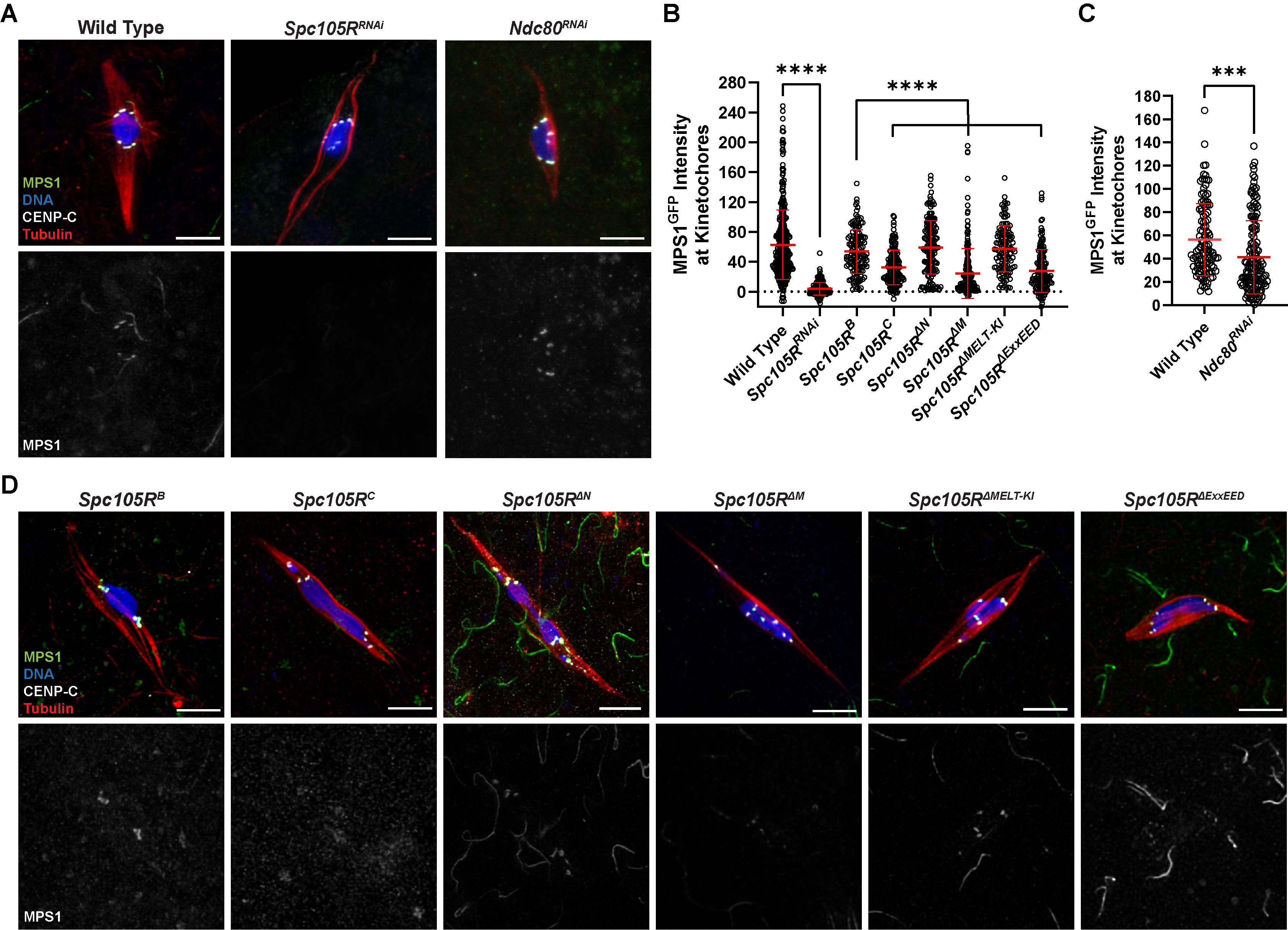

Supplement: S4 Fig — (A) MPS1GFP localization in wild-type, Spc105RRNAi, and Ndc80RNAi oocytes, with MPS1GFP in green, DNA in blue, CENP-C in white, and tubulin in red. Single channel images (bottom) show MPS1GFP. (B) Quantification of MPS1GFP intensity at kinetochores, normalized to background GFP signal in indicated oocytes (from left to right, n = 389, 195, 139, 160, 165, 198, 128, and 143 kinetochores). Error bars show mean ± s.d.; ****P<0.0001 (unpaired two-tailed t test). (C) Quantification of MPS1GFP intensity at kinetochores, normalized to background GFP signal in wild-type and Ndc80RNAi oocytes (n = 114 and 148 kinetochores). Error bars show mean ± s.d.; ****P<0.0001 (unpaired two-tailed t test). (D) MPS1GFP localization in the indicated Spc105R mutants with MPS1GFP in green, DNA in blue, CENP-C in white, and tubulin in red. All mutants are in an Spc105RRNAi background targeting the endogenous Spc105R. Single channel images (bottom) show MPS1GFP. All images are maximum intensity projections of z stacks. Scale bars represent 5 μm. (TIF) [file pgen.1011400.s004.tif]

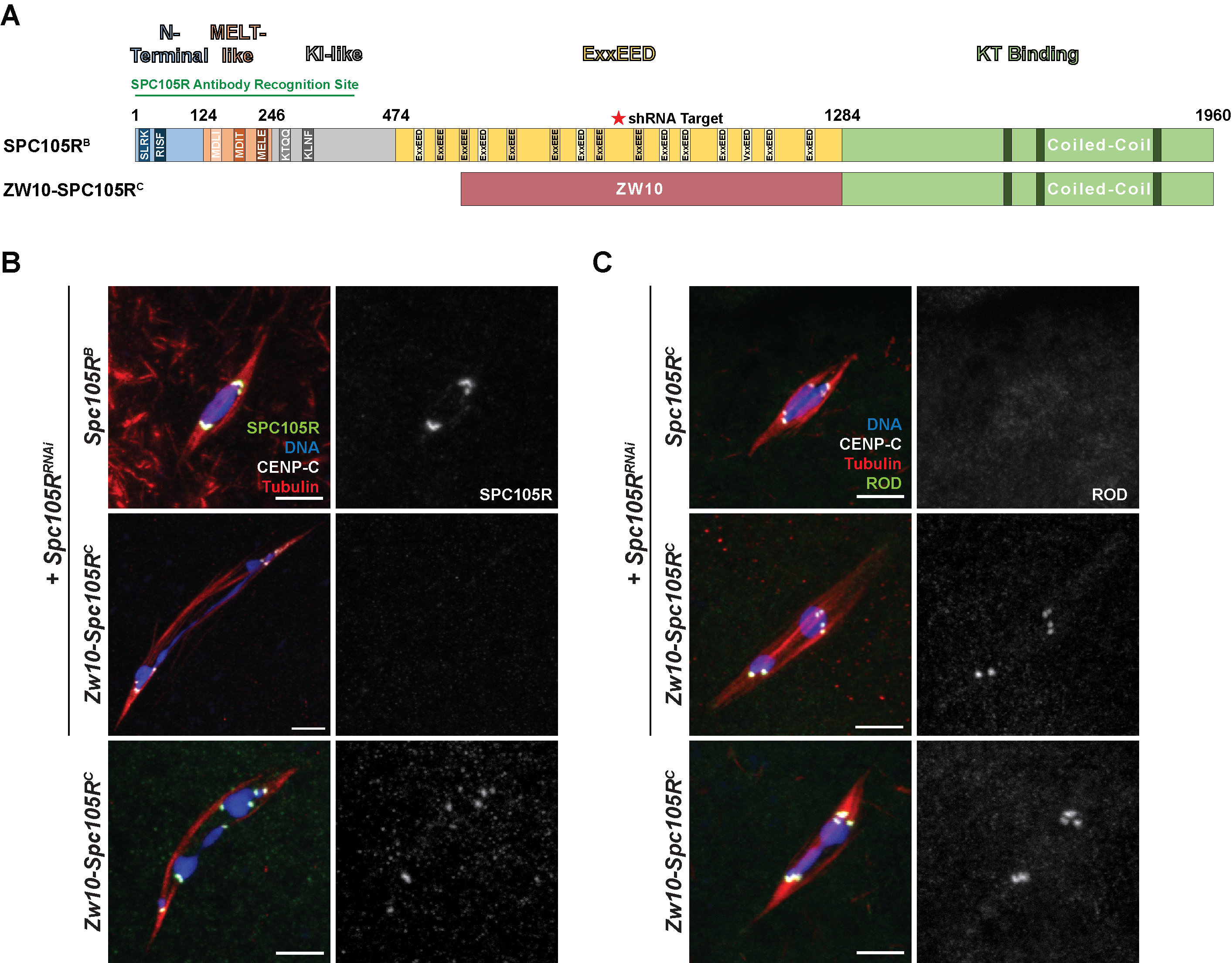

Supplement: S5 Fig — (A) Schematic of SPC105RB and the ZW10-SPC105RC fusion. (B) SPC105R localization in Zw10-Spc105RC, either in the presence or absence of Spc105RRNAi. SPC105R (green) was detected using an antibody which recognizes the N-terminal regions of SPC105R and does not detect SPC105RC. DNA is in blue, CENP-C in white, and tubulin in red. (C) RODGFP localization in oocytes expressing Zw10-Spc105RC, either in the presence or absence of Spc105RRNAi. RODGFP is in green, DNA in blue, CENP-C in white, and tubulin in red. All images are maximum intensity projections of z stacks. Scale bars represent 5 μm. (TIF) [file pgen.1011400.s005.tif]

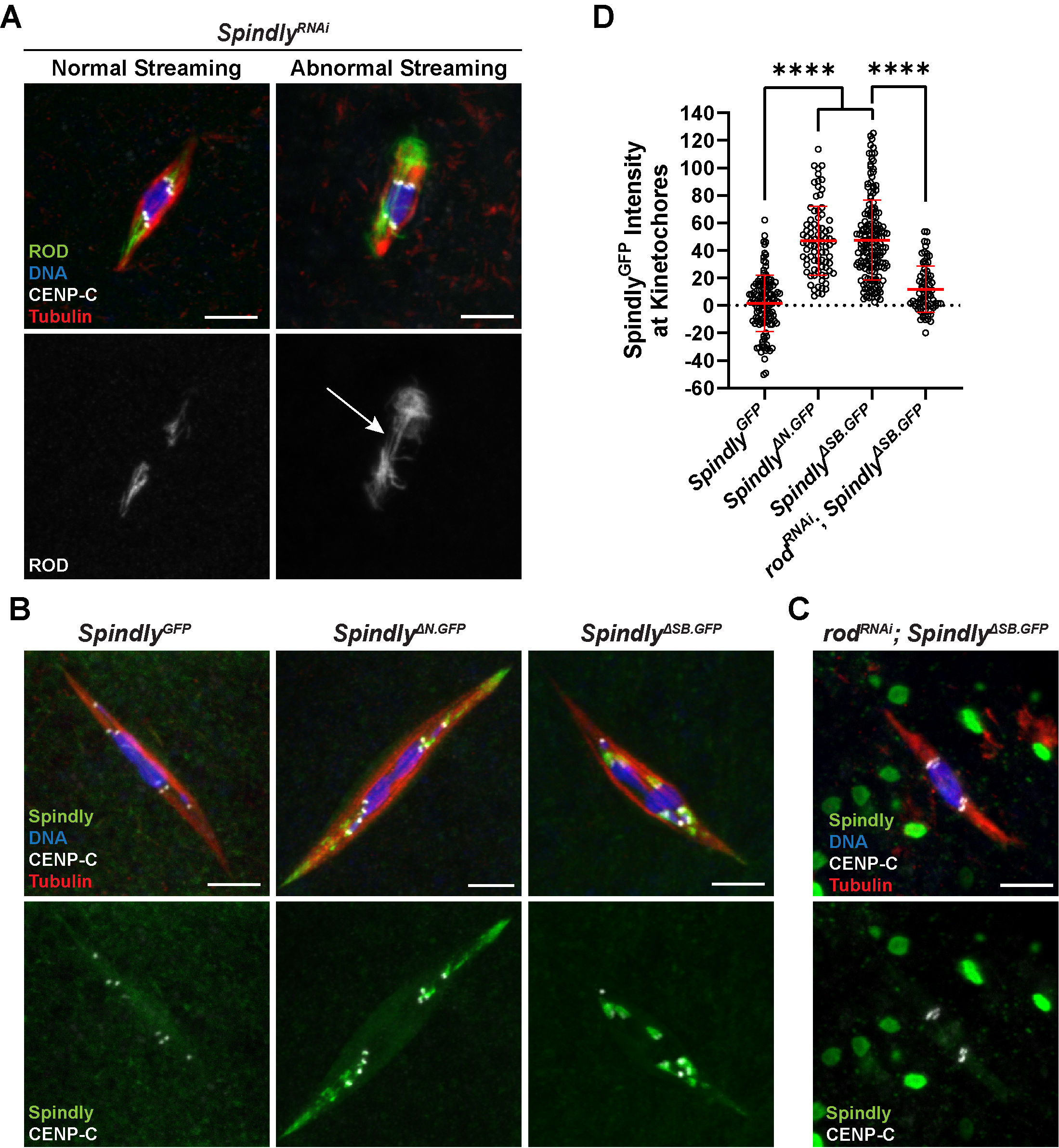

Supplement: S6 Fig — (A) Examples of normal (left) and abnormal (right) RODGFP streaming in SpindlyRNAi oocytes, with RODGFP in green, DNA in blue, CENP-C in white, and tubulin in red. Single channel images (bottom) show RODGFP. Arrow points to abnormal streaming, which is defined as having RODGFP in the central region between the centromeres. (B) SpindlyGFP localization in wild-type or mutants of Spindly. SpindlyGFP is in green, DNA in blue, CENP-C in white, and tubulin in red. (C) Localization of SpindlyΔSB.GFP in rodRNAi oocytes, with SpindlyGFP in green, DNA in blue, CENP-C in white, and tubulin in red. SpindlyGFP and CENP-C are shown below the merged images. All images are maximum intensity projections of z stacks. Scale bars represent 5 μm. (D) Quantification of SpindlyGFP intensity at kinetochores in the indicated oocytes (from left to right, n = 123, 164, 83, and 75 kinetochores). Error bars show mean ± s.d.; ****P<0.0001 (unpaired two-tailed t test). (TIF) [file pgen.1011400.s006.tif]
