# Supplementary material for: Distinct checkpoint and homolog biorientation pathways regulate meiosis I in Drosophila oocytes
Source: PLoS Genet. 2025 Jan 29;21(1):e1011400. doi: 10.1371/journal.pgen.1011400 (PMC11809923; doi:10.1371/journal.pgen.1011400)
Supplement: S1 Table — (DOCX) [file pgen.1011400.s007.docx]

Table S 1: Summary of SAC protein localization data

|  | **ROD** | **MPS1** | **INCENP*** | **MAD1*** |
| --- | --- | --- | --- | --- |
| *Spc105R^RNAi^* | 0 | 0 | 0 | − |
| *Spc105R^ΔN^* | ++ | + | N/A | N/A |
| *Spc105R^ΔMELT^* | − | N/A | N/A | N/A |
| *Spc105R^ΔKI^* | − | N/A | N/A | N/A |
| *Spc105R^ΔExxEED^* | ++ | − | + | − |
| *Spc105R^ΔMELT-KI^* | − | + | + | + |
| *Spc105R^ΔM^* | 0 | − | − | − |
| *Spc105R^C^* | 0 | − | − | − |

*measured in colchicine-treated oocytes

0 = little to no protein at kinetochores

− = significantly reduced protein at kinetochores

+ = protein levels at kinetochores similar to *Spc105R^B^*

++ = significantly increased protein at kinetochores

N/A = protein levels not measured
