# Supplementary material for: Distinct checkpoint and homolog biorientation pathways regulate meiosis I in Drosophila oocytes
Source: PLoS Genet. 2025 Jan 29;21(1):e1011400. doi: 10.1371/journal.pgen.1011400 (PMC11809923; doi:10.1371/journal.pgen.1011400)
Supplement: S2 Table — (DOCX) [file pgen.1011400.s008.docx]

Table S 2: Meiotic X-chromosome nondisjunction in *rod* and *mps1* transgenes

| **Genotype** | **XX** | **XY** | **XXY** | **XO** | **% NDJ** |
| --- | --- | --- | --- | --- | --- |
| *w^1118^/matα* | 351 | 317 | 0 | 1 | 0.30 |
| *w^1118^/rod^GFP^matα* | 363 | 389 | 0 | 1 | 0.27 |
| *w^1118^/mps1^GFP^matα* | 172 | 149 | 0 | 0 | 0.00 |

Genotypes refer to the gametes from the female parent.

NDJ = nondisjunction
